# NTI164, a novel medicinal cannabis extract, improves core symptoms of autism spectrum disorder: Results from a double-blind, randomised, controlled trial (The Harmony study)

**DOI:** 10.1016/j.neurot.2026.e01033

**Published:** 2026-08-06

**Authors:** Brooke A. Keating, Yelda Ogru, Esra Isikgel, Kanan Sharma, Dima El-Sukkari, Michael C. Fahey

**Affiliations:** a Fenix Innovation Group, Mount Waverley, Victoria, Australia; b Monash Children's Clinical Trial Centre, Monash Children's Hospital, Monash Health, Clayton, Victoria, Australia; c Department of Neurology, Monash Children's Hospital, Monash Health, Clayton, Victoria, Australia

**Keywords:** Cannabis, Autism, NTI164, Social responsiveness, Mood

## Abstract

Autism spectrum disorder (ASD) is a heterogeneous neurodevelopmental disorder, characterised by difficulties with communication, social interaction, repetitive behaviours, restricted interests, and varying levels of intellectual disability. Aetiology remains unclear for many patients and the underlying physiology is complex. Approved pharmacological treatments of ASD target irritability, temper tantrums, and agitation, with no therapies targeting core ASD symptoms. This double-blind, randomised, placebo-controlled Phase II/III clinical trial investigated the efficacy and safety of NTI164, a novel full-spectrum medicinal cannabis product with <0.3% tetrahydrocannabinol (THC), in paediatric patients with Level II/III ASD. Participants were recruited from a tertiary paediatric neurology clinic and randomised to receive NTI164 up to 20 mg/kg/day or placebo for an 8-week double-blind phase; participants receiving placebo were able to receive NTI164 in an 8-week open label phase following the double-blind phase. Safety assessments, clinician-, and caregiver-rated tools measuring symptoms were utilised at baseline and Week 8. Analysis of Covariance (ANCOVA) was used for statistical analyses. NTI164 demonstrated an excellent safety profile, and statistically significant and meaningful improvements compared to placebo in overall clinical severity, adaptive functioning, social responsiveness, and affective symptoms. Caregivers also reported improved family experiences and quality of life with NTI164. Participants who transitioned from placebo to NTI164 open label reported similar improvements as those reported during the double-blind phase. NTI164 significantly improved core and associated symptoms of ASD compared to placebo. Consistent benefits reported by both clinicians and caregivers in both open label and double-blind contexts supports further clinical development of NTI164 in ASD.

## Introduction

Autism spectrum disorder (ASD) is a clinically heterogeneous neurodevelopmental disorder (NDD), with a worldwide prevalence estimated at ∼1%, with estimates higher in higher-income countries [1]. ASD often presents as impairments in social interaction and communication, sensory abnormalities, repetitive behaviours, restricted interests, and varying degrees of intellectual disability. Co-morbid neurological and psychiatric conditions are prevalent in ASD patients, particularly anxiety, depression, obsessive-compulsive disorder (OCD), attention deficit hyperactivity disorder (ADHD), and epilepsy [2]. In Australia, ASD and its associated symptoms are estimated to cost $4.5–7.2 billion, a cost borne by individuals, families, and government, and costs are ultimately lifelong [3].

ASD is considered a complex genetic disorder with high heritability, with studies of heterozygous, germline, *de novo* mutations identifying >100 genes associated with ASD [4,5]. However, a significant proportion of cases have no identified genetic cause, and it is now widely accepted underlying epigenetic modifications and environmental interactions, including advanced parental age, pregnancy-related complications, maternal inflammation during pregnancy, and exposure to toxins, contribute to ASD development in a subset of patients [6,7].

The pathophysiology of ASD is similarly complex, with some studies showing volumetric differences in specific brain regions in young individuals with ASD compared to those without [8], however synaptic dysfunction is often labelled the hallmark feature of ASD pathophysiology [9]. Many known high-risk genes linked to ASD encode for proteins related to synaptic function in the brain [10], including protein synthesis and degradation, and chromatin remodelling, affecting synaptic homeostasis and neuroplasticity [[11], [12], [13]]. Studies have also implicated physiological and metabolic abnormalities in the pathophysiology of ASD, including oxidative stress, aberrant inflammation, and mitochondrial dysfunction [14]. Many studies have also demonstrated shared mechanistic abnormalities between ASD and common comorbidities, including other NDDs and neuropsychiatric conditions [14,15].

Intervention for ASD patients often involves both psychological and pharmacological approaches. Psychological, occupational, and physical therapies can be useful to manage and improve symptoms around anxiety, communication, daily living skills, and repetitive behaviours, while medications only target specific symptoms [16]. The atypical antipsychotics risperidone and aripiprazole are the only FDA-approved medications for use in ASD, however both are only registered for irritability [17], with limited options available to patients and families for other problematic symptoms and behaviours. These medications also commonly produce troubling side effects which can limit clinical utility for many patients, including sedation, significant weight gain, and constipation [18].

The number of children receiving diagnoses of ASD has been argued to be steadily increasing, with the reason behind this unclear [19]. While some argue an increase in patient numbers due to improved diagnostic procedures, this does not account for the entirety of the increase observed and there seems to be a genuine medical reason underlying the increase in numbers [20], and effective symptom management is essential for both patients and families.

*Cannabis sativa* L. has been used in medicine for millennia, with use as an adjunct therapy in psychiatric disorders and NDDs increasing in recent decades [21]. The cannabis plant secretes a resin containing a variety of cannabinoids with two major compounds, Δ9-tetrahydrocannabinol (THC) and cannabidiol (CBD). CBD has been the focus of extensive clinical research due to an absence of psychoactivity, potent anti-inflammatory capabilities, and excellent tolerability in humans [22], while THC is known to also elicit clinically beneficial results in many contexts but its innate psychoactivity limiting clinical utility [23]. Increasing volumes of data suggest full-spectrum cannabis products (i.e. all cannabinoids are present) are more potent immunomodulators and anti-inflammatory than isolated compounds [24,25], and as such low-THC cannabis products are increasing in popularity across many medical fields, including neurology and psychiatry.

This study builds on evidence of efficacy of NTI164 in ASD from a Phase I/II study [26]. In this double-blind, randomised, placebo-controlled clinical trial (NTIASD2, ClinicalTrials.gov ID: NCT05626959), we investigated the safety and efficacy of NTI164, a standardised full-spectrum medicinal cannabis plant extract derived from proprietary strains of *Cannabis sativa* on improving symptoms of ASD compared to placebo. NTI164 contains a blend of cannabidolic acid (CBDA), CBD, cannabigerolic acid (CBGA), cannabidivarian (CBDV), and extremely low levels of THC (<0.3%), and has shown efficacy in several open-label clinical trials in other paediatric neurological conditions (Clinical.Trials.gov IDs: NCT06621043, NCT06621888). NTI164 or placebo was administered orally twice daily for 8 weeks at doses up to 20 mg/kg/day, with clinician- and caregiver-rated assessments relating to clinical severity and symptoms performed at baseline (prior to treatment allocation) and at Week 8 (primary endpoint). Clinical pathology was also performed to expand existing safety data relating to NTI164.

## Materials and methods

### Ethics approval and informed consent

Ethics approval for this study was granted by Monash Health Human Research Ethics Committee, Victoria, Australia (HREC reference: RES-22-0000-592A). All participants and their parent/legal guardian provided written informed consent for participation in this study. This study was conducted in accordance with ICH-GCP guidelines, and federal and local governing regulatory requirements.

### Participant selection

Children were recruited from specialist neurology clinics at Monash Children's Hospital, and all participants had an existing diagnosis of either Level II or III ASD (moderate-severe). See Supplementary Material, Appendix 1, for full inclusion/exclusion criteria.

### Study design

Sixty-one (61) participants fulfilling inclusion criteria were enrolled into the study and randomly allocated in a 1:1 ratio to either the Active (NTI164) or Placebo group, according to a list generated by GraphPad Randomised Assignment Generator allocating participants to either Group A (Active) or Group B (Placebo). The study involved an 8-week double-blind phase during which only hospital Pharmacy staff had access to patient group allocation in case emergency unblinding was required. No patient required emergency unblinding during the study. The placebo suspension was visually indistinguishable from NTI164 and packaged identically.

The sample size of this study was calculated based on assumptions from a previous clinical trial of NTI164 in ASD Level II/III [26]. It was assumed the average difference in change from baseline of the CGI-S score between the Active and Placebo groups is -0.9 points with a common standard deviation of 1, and that the mean CGI-S score at Week 8 for the Placebo group is 4.0 (no change) and 3.0 or less (improved) for the Active group. Using a two-sided *t*-test at a 5% significance level and a desired power of 90%, the required sample size to detect a treatment effect of -0.9 is 54 participants (27 per arm).

Simple randomisation was considered appropriate for this study population as participants with ASD Level II or III generally share comparable core diagnostic features, levels of functional impairment, and clinical support needs. Eligibility criteria were also designed to enrol a relatively homogeneous population with respect to disease severity (although co-occurring Intellectual Development Disorder information was not specifically captured), while concomitant medication use and background therapies were expected to be similarly distributed between treatment groups, even with simple randomisation. Analyses at the conclusion of the study period supported these assumptions, and there were no significant differences in mean CGI-S scores at baseline between groups, or the mean number of concomitant medications. This study involved an initial 8-week double-blind phase, followed by an 8-week open-label phase. For the first 4-weeks of the double-blind phase, all participants underwent up-titration from a starting dose of 5 mg/kg/day in Week 1 which increased weekly by 5 mg/kg/week, to a targeted maximum tolerated dose of 20 mg/kg/day by Week 4. The Placebo group received matched volumes of the placebo suspension.

At Week 8, all participants were unblinded and given the opportunity to receive NTI164 for an additional 8 weeks in an open-label phase. For participants who had received placebo during the double-blind phase, the same 4-week up-titration protocol was followed with patients receiving 20 mg/kg/day (or their maximum tolerated dose as determined by their treating clinician) by Week 12.

At the end of the study (Week 16) patients wanting to remain on NTI164 were able to do so and entered an ongoing extension phase (n = 44).

### Questionnaires

Questionnaires were administered by trained personnel at baseline (prior to treatment), Week 4, Week 8 (end of double-blind phase), and Week 16 (end of open label phase). See Table 1 for details on questionnaires used. Parent/caregiver-rated questionnaires were completed at the same time points in the presence of study staff. Staff administering questionnaires up to and including Week 8 were blinded to treatment group allocation of participants.

**Table 1:** Questionnaires used in the NTIASD2 study.

Questionnaire	Assesses	Timepoints assessed
Primary outcome Clinical Global Impression – Severity (CGI-S) *Clinician-rated*	Reflects clinicians' impression of severity of illness at a point in time on a 7-point scale ranging from 1 = not at all to 7 = among the most extremely ill A higher score indicates worse/more severe condition	All surveys performed at baseline (prior to treatment), Week 4, Week 8, Week 16
Clinical Global Impression – Improvement *Clinician-rated*	Reflects clinicians' impression of degree of improvement following an intervention on a 7-point scale with 1 = very much improved, 4 = no change, and 7 = very much worse A higher score indicates less improvement/worsening	
Vineland Adaptive Behaviour Scales, Third Edition (Vineland-3) *Clinician-rated*	Used to measure adaptive functioning across three core domains: Communication, Daily Living Skills, and Socialisation. Items are rated on a 3-point scale: 0 = never, 1 = sometimes, 2 = usually or often. The core domains sum to a total Adaptive Behaviour Composite. A higher score indicates better functioning	
Social Responsiveness Scale, Second Edition (SRS-2) *Caregiver-rated*	Assesses five domains: Social Awareness, Social Cognition, Social Communication, Social Motivation, and Restricted Interests and Repetitive Behaviour. Items are scored on a 4-point scale with 1 = not true to 4 = almost always true. A higher score indicates greater impairment.	
Anxiety, Depression, and Mood Scale (ADAMS) *Caregiver-rated*	Assesses five subdomains: Manic/hyperactive Behaviour, Depressed Mood, Social Avoidance, General Anxiety, and Compulsive Behaviour. Items are rated on a 4-point scale ranging from 0 = not a problem to 3 = severe problem Higher score indicates greater impairment	
Autism Family Experience Questionnaire (AFEQ) *Caregiver-rated*	Measures the impact of autism interventions on family experience and quality of life. Items are rated on a 5-point scale where 1 = always and 5 = never. Higher score indicates poorer outcomes.	
Anxiety Scale for Children – ASD – Parent version (ASC-ASD-P) *Caregiver-rated*	Detects symptoms of anxiety in youth with ASD. Composed of four subdomains: Performance Anxiety, Uncertainty, Anxious Arousal, and Separation Anxiety. Items are rated on a 4-point scale with 0 = never to 3 = always. Subdomains sum to a Total Score. Higher score indicates greater anxiety	
Sleep Disturbance Scale for Children (SDSC) *Caregiver-rated*	Assesses six subdomains: Disorders of Initiating and Maintaining Sleep, Sleep Breathing Disorders, Disorders of Arousal, Sleep Wake Transition Disorders, Disorders of Excessive Somnolence, and Sleep Hyperhidrosis. Items are rated on a 5-point scale where 1 = never and 5 = always. Subdomains sum to a Total Score.	

### Safety bloods

Venous blood was collected by qualified staff for all participants prior to commencing treatment as well as at Week 4, Week 8, and Week 16. See Supplementary Table 1 for a detailed list of parameters monitored. If, at the discretion of the treating clinician, a patient required sedation for blood collection, this was arranged with the appropriate hospital ward and blood collected under sedation with nitrous oxide in oxygen.

### Statistical analysis

All statistical analyses up to and including Week 8 were conducted by an independent statistician blinded to treatment groups. Analyses were performed using R (v4.2.2).

The primary efficacy outcome assessed the change from baseline to Week 8 in the CGI-S score, comparing NTI164 with placebo. Analysis was conducted using Analysis of Covariance (ANCOVA), adjusting for baseline CGI-S as a covariate. Least squares means and 95% confidence intervals were presented for each treatment group, along with the estimated between-group difference.

Secondary outcomes were also assessed using ANCOVA.

Safety data, including AEs, laboratory results, vital signs, and physical examinations, were summarised descriptively. AEs were coded using the MedDRA and tabulated by system organ class and preferred term, severity, and relationship to study treatment.

All statistical tests were two-sided with a significance level of α = 0.05. Confidence intervals were presented at the 95% level, and no adjustment for multiplicity was applied to secondary outcomes.

Exploratory analyses were performed for participants who received placebo during the double-blind phase and subsequently transitioned to NTI164 during the open label phase. These analyses evaluated within-subject changes from Week 8 (end of placebo) to Week 16 (8 weeks on NTI164). Results were presented as descriptive statistics, with paired t-tests applied for continuous endpoints and Wilcoxon signed-rank tests used where parametric assumptions were not met. Effect sizes and corresponding 95% confidence intervals were provided for each endpoint, and all tests were two-sided.

## Results

### Clinical characteristics of participants

A total of sixty-one (61) participants with an existing Level II or III (moderate-severe) ASD diagnosis from Victoria, Australia, were enrolled into this study from a tertiary paediatric neurology clinic, randomly allocated in a 1:1 ratio to either the Active Group (NTI164) or Placebo, and received at least one dose of study drug. Demographic and baseline clinical characteristics were comparable across groups (Table 2). The mean age of participants was 12.2 years (range: 8–17 years), and 29 participants were male (53.7%).

Data relating to seizures, IQ scores, and genetic screening for ASD-linked genetic variants was not collected.

At the time of enrolment, children were taking an average of 2.85 medications (no difference across treatment groups), including α-agonists (n = 22, 40.7%), central nervous system (CNS) stimulants (n = 19, 35.2%), CNS depressants (n = 18, 33.3%), antipsychotics (n = 16, 29.6%), and selective serotonin reuptake inhibitors (SSRIs; n = 13, 24.1%). As part of inclusion criteria into the current study, all medications were required to be at a stable dose (i.e. unchanged) for at least 12-weeks prior to enrolment.

### Severity of ASD symptoms

The Clinical Global Impression – Severity (CGI-S; primary endpoint for the study) was used to assess the severity of illness at baseline (i.e. prior to treatment commencement). Baseline CGI-S scores were comparable between treatment groups (Table 2), with a mean baseline CGI-S score for the Active group of 5.5 and a mean score of 5.2 for Placebo. Overall, enrolled participants had a mean baseline CGI-S score of 5.4, with values ranging from 3 to 7 across both treatment arms. These scores indicate most participants were rated as *markedly to severely ill* at the time of commencing treatment.

**Table 2:** Baseline demographic and clinical characteristics of participants in the Harmony study.

**Characteristic**	**NTI164 (n = 26)**	**Placebo (n = 28)**	**Total (n = 54)**
**Age (years)**			
Mean (±SD)	12.38 ± 2.26	11.96 ± 2.20	12.17 ± 2.22
Median (Min-Max)	12.0 (9–16)	11.5 (8–17)	12.0 (8–17)
**Sex, n (%)**			
Male	14 (53.8%)	15 (53.6%)	29 (53.7%)
Female	12 (46.2%)	13 (46.4%)	25 (46.3%)
**CGI-S Severity of Illness**			
Mean (±SD)	5.54 ± 1.07	5.21 ± 1.17	5.37 ± 1.12
Range (Min-Max)	3-7	3-7	3-7

### Safety and tolerability

Of the 61 enrolled participants, 54 participants (88.5%) completed the 8-week double-blind phase (n = 26 Active, n = 28 Placebo). The seven participants (11.5%) who discontinued prior to completion of the double-blind phase discontinued due to taste intolerance (n = 4), oil intolerance (n = 1), withdrawal of consent (n = 1), and one adverse event (AE) considered unrelated to the study drug (n = 1, emergency unblinding not required).

A total of 41 AEs were reported by 26 participants over the 8-week double-blind phase of this study. All AEs were determined to be mild, non-serious, transient, and self-resolving. No serious adverse events (SAEs) were reported over the course of the study. Most reported AEs (n = 30, 73.2%) were transient gastrointestinal and/or neurological symptoms, with nausea (n = 9, 21.9%), headache (n = 8, 19.5%), abdominal pain (n = 7, 17.1%), and anxiety (n = 6, 14.6%) being the most frequently observed. See Supplementary Table 2 for further details on AEs reported during the study. These AEs were evenly distributed across study groups. Patient doses ranged from 10 to 20 mg/kg/day during the 8-week double-blind period.

No clinically significant changes were observed in clinical pathology laboratory values, or vital signs, at any point during the study period. See Supplementary Table 1 for a full list of parameters monitored during the study period. NTI164 was confirmed to have a favourable safety and tolerability profile in this paediatric ASD population, with a safety profile comparable to placebo. Following completion of the double-blind phase, 44 participants entered the open-label extension/maintenance phase (n = 29 from Placebo and 19 from Active). Reasons for participants discontinuing following the main study period included taste (n = 6), travel (n = 2), and lost to follow up (n = 2).

### NTI164 improved overall clinical impression of disease severity compared to placebo

The primary outcome of the current study was the CGI-S, a 7-point scale of a clinician's impression of disease severity at a given timepoint. Scores were generated at baseline (i.e. prior to commencing NTI164 or Placebo), at Week 4 (i.e. end of up-titration phase), and at Week 8 (primary endpoint).

**Fig. 1:**
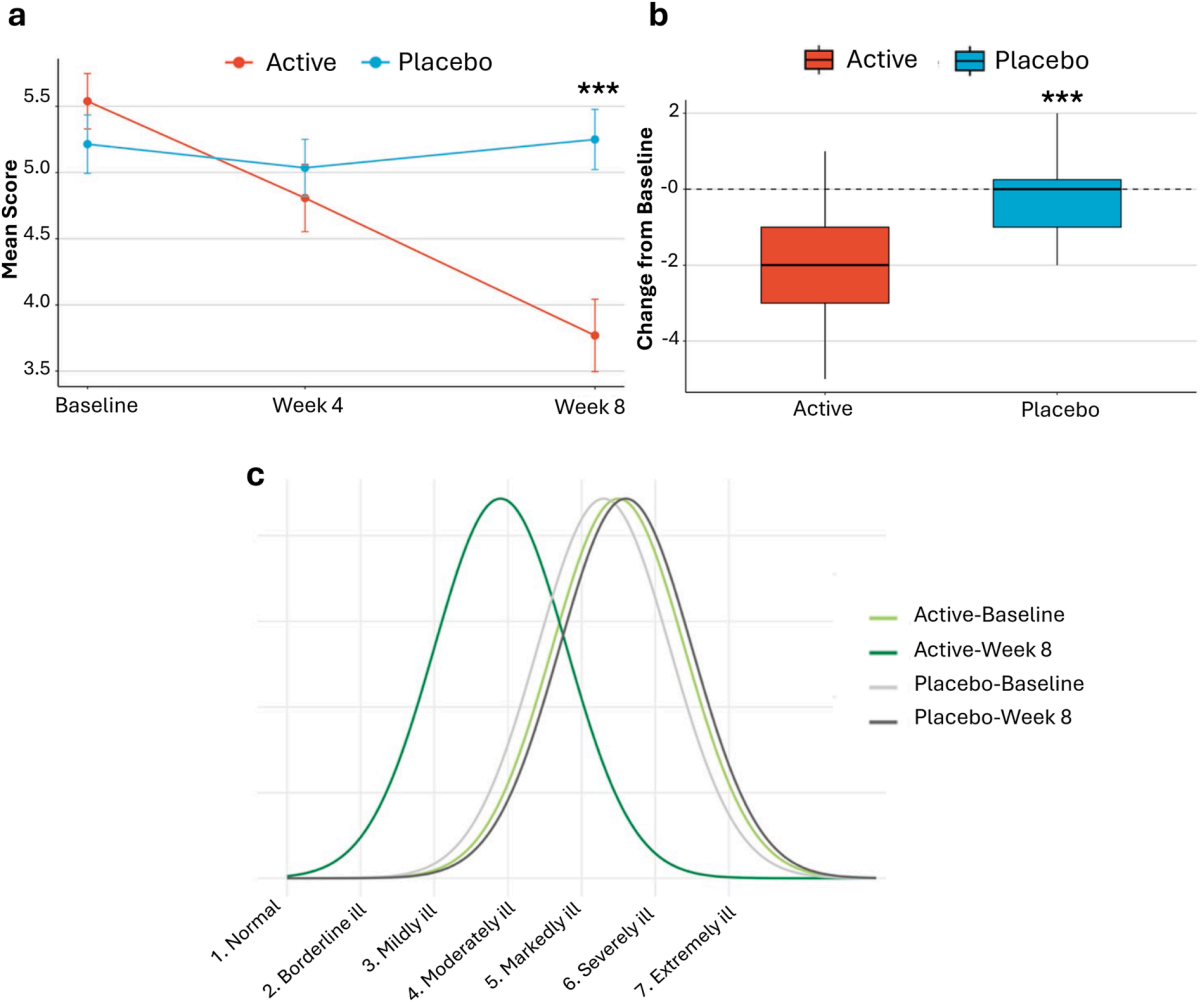
**NTI164 significantly improved clinical impressions of severity (CGI-S) compared to placebo** (a) Mean ± change from baseline in CGI-S scores over time (b) Distribution of change from baseline in CGI-S scores at Week 8 (c) Modelled distribution of CGI-S scores over time by treatment group and timepoint. n = 54, analysed via ANCOVA, adjusting for baseline CGI-S as a covariate, ∗∗∗p < 0.001.

No significant difference of baseline scores was observed between groups, however separation between groups was evident at both Week 4 and Week 8 (Fig. 1A). ANCOVA analysis showed a least-squares (LS) mean (± standard error (SE)) difference between NTI164 and Placebo of -1.65 (0.32), 95% CI: -2.29 to -1.01; p < 0.001, confirming a statistically significant treatment effect in favour of NTI164 over placebo (Fig. 1B). The estimated effect size corresponds to an approximate one-category improvement on the 7-point CGI-S scale (Fig. 1C). See Supplementary Table 3 for descriptive statistics of the change between groups.

### NTI164 improved clinical impressions of symptom improvement compared to placebo

**Fig. 2:**
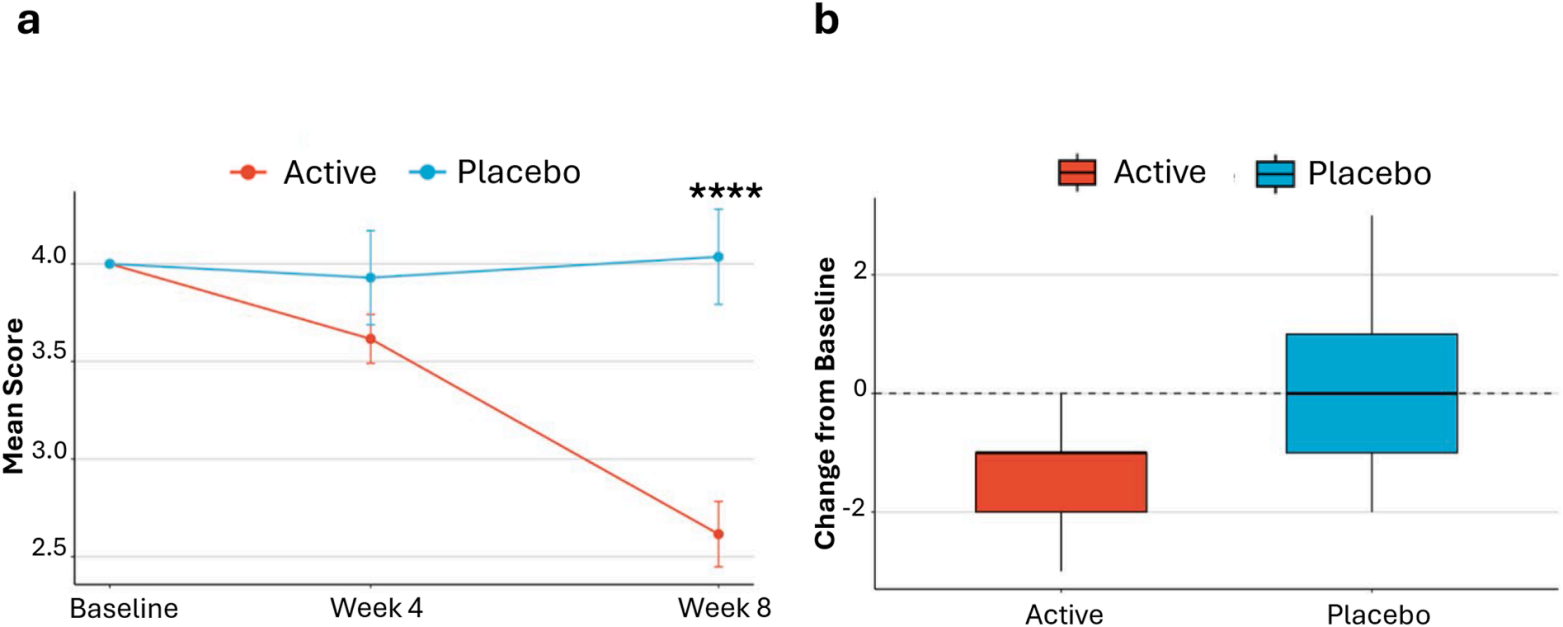
**NTI164 significantly improved clinical impressions of symptoms of ASD compared to placebo** (a) Mean ± change from baseline in total CGI-I scores over time. (b) Distribution of change from baseline in CGI-I scores at Week 8. n = 54, analysed via ANCOVA, adjusting for baseline CGI-I as a covariate, ∗∗∗∗p < 0.0001.

At Week 8, patients treated with NTI164 demonstrated greater clinical improvement compared with those receiving placebo, indicated by lower mean CGI-I scores (Fig. 2A). Participants were assigned a reference value of 4 (“no change”) for the purpose of deriving a descriptive improvement score, calculated as 4 minus the Week 8 CGI-I score. The mean CGI-I score at Week 8 was 2.62 for participants receiving NTI164, and 4.04 for participants receiving placebo, reflecting a mean score of between “minimally improved’ and “much improved” (3-2, respectively) for the Active group compared to a mean score reflecting “no change” [4] in the Placebo group,

supporting the overall direction of benefit observed in the primary efficacy endpoint. The mean ± standard deviation (SD) change in scores at Week 8 was -1.38 ± 0.85 for patients receiving NTI164, compared with 0.04 ± 1.29 for patients receiving placebo. See Supplementary Table 4 for further descriptive statistics of change from baseline between treatment groups.

### NTI164 improved adaptive functioning in ASD compared to placebo

The Vineland Adaptive Behaviour Scales, Third Edition (Vineland-3) measures functioning across three core domains: Communication, Daily Living Skills, and Socialisation. Scores across the core domains sum to a total Adaptive Behaviour Composite (ABC). Change from baseline in Vineland-3 scores across the core domains and ABC were calculated using an ANCOVA model with treatment group as a fixed effect and baseline score as a covariate.

**Fig. 3:**
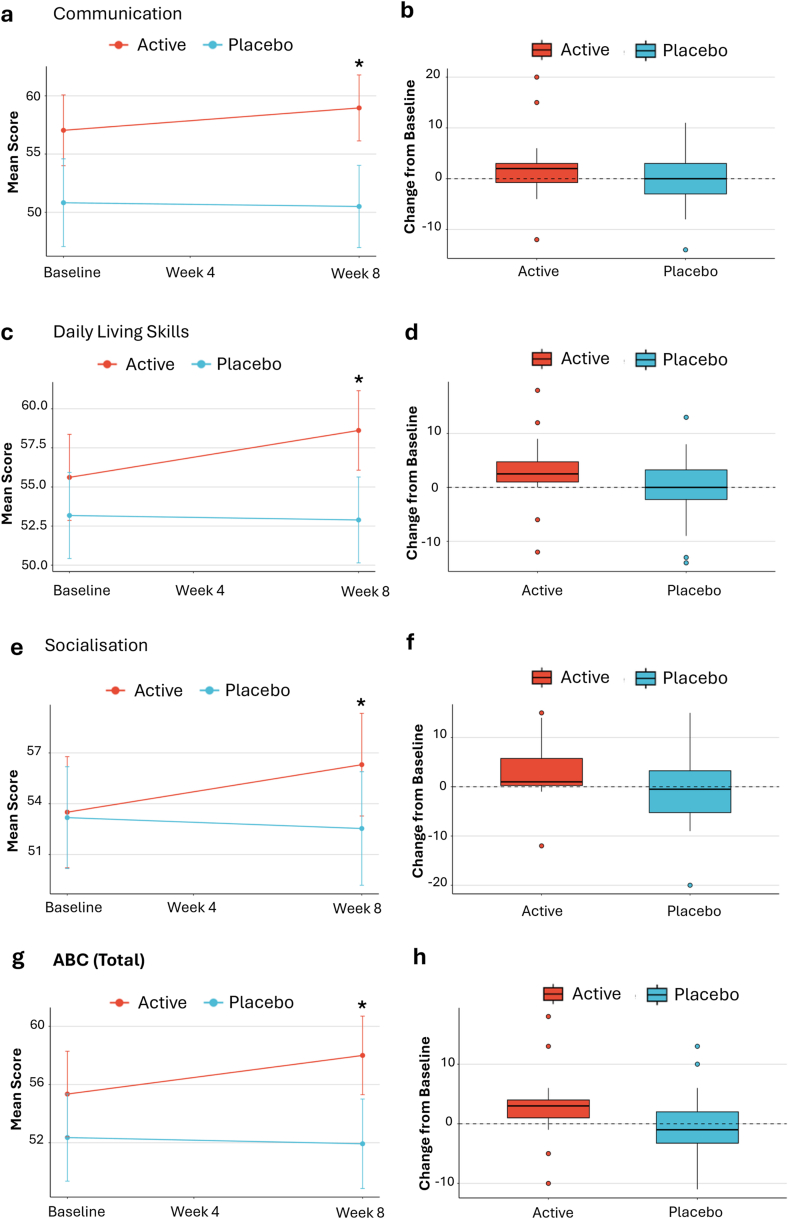
**NTI164 improved adaptive functioning in ASD compared to placebo, as measured using the Vineland-3** (a) Mean ± change from baseline in Communication scores over time. (b) Distribution of change from baseline in Communication scores at Week 8. (c) Mean ± change from baseline in Daily Living Skills scores over time. (d) Distribution of change from baseline in Daily Living Skills scores at Week 8. (e) Mean ± change from baseline in Socialisation scores over time. (f) Distribution of change from baseline in Socialisation scores at Week 8. (g) Mean ± change from baseline in Total ABC scores over time. (h) Distribution of change from baseline in Total ABC scores at Week 8. n = 54, analysed via ANCOVA, ∗p < 0.05.

At Week 8, participants receiving NTI164 demonstrated greater improvement compared with placebo across all Vineland-3 core domains and ABC (Fig. 3A–C, E, G). The LS mean difference (±SE) for Communication was 2.92 (±1.43), p = 0.047, for Daily Living Skills was 3.56 (±1.50), p = 0.021, for Socialisation was 3.47 (±1.71), p = 0.048, and for ABC was 3.31 (±1.39), p = 0.021. Across all domains and ABC, mean (±SE) scores for the NTI164 group increased from baseline to Week 8 (i.e. improve), whereas scores in the placebo group either remained stable or slightly declined (i.e. worsened). Boxplots of change-from-baseline distributions illustrate these improvements compared to baseline at Week 8 over placebo (Fig. 3B–D, F, H).

These findings indicate NTI164 produced statistically significant and clinically meaningful improvements in adaptive behaviour, communication, daily living skills, and socialisation compared with placebo over the 8-week double-blind period. See Supplementary Table 5 for further descriptive statistics of change from baseline between treatment groups.

### NTI164 is associated with favourable changes in behaviours across domains of socialness

The Social Responsiveness Scale, Second Edition (SRS-2) assesses five domains: Social Awareness, Social Cognition, Social Communication, Social Motivation, and Restricted Interests and Repetitive Behaviour, with overall scores presented as a Total T-score.

**Fig. 4:**
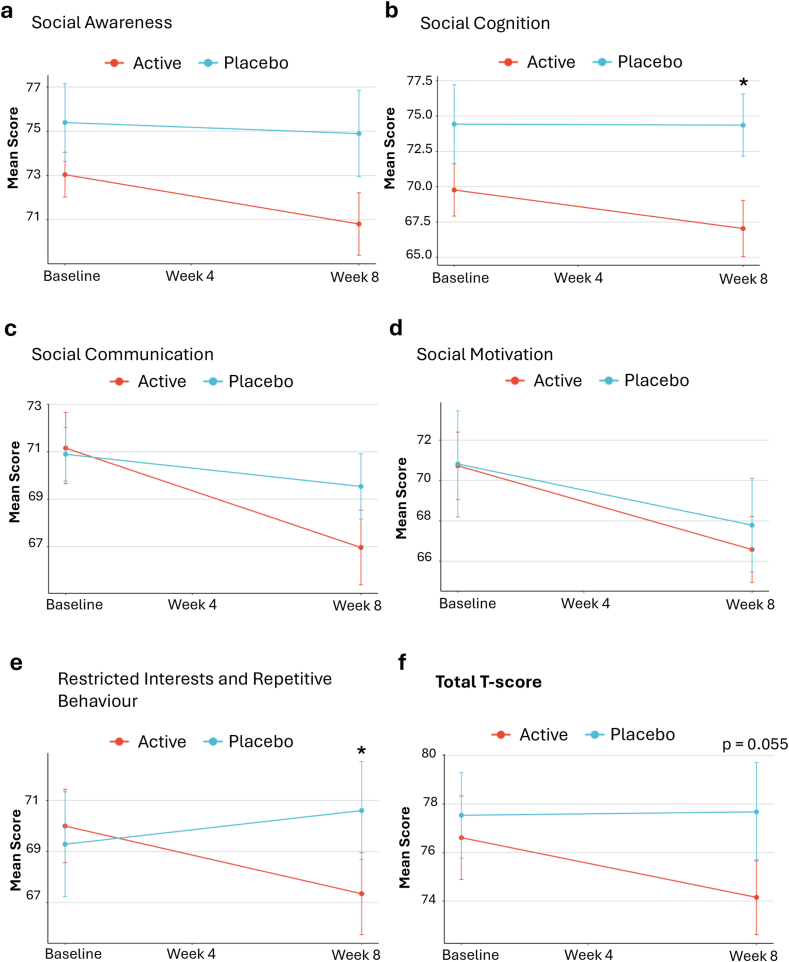
**NTI164 improved behaviours across domains of social responsiveness, as measured by the SRS-2** (a) Mean ± change from baseline in Social Awareness scores over time. (b) Mean ± change from baseline in Social Cognition scores over time. (c) Mean ± change from baseline in Social Communication scores over time. (d) Mean ± change from baseline in Social Motivation scores over time. (e) Mean ± change from baseline in Restricted Interests and Repetitive Behaviour scores over time. (f) Mean ± change from baseline in Total T-scores over time. n = 54, analysed via ANCOVA, ∗p < 0.05.

Assessment of social responsiveness using the SRS-2 demonstrated favourable trends in overall social functioning following 8 weeks of oral administration of NTI164 compared to placebo (Fig. 4). Consistent reductions were observed across several SRS-2 subdomains, particularly in Social Cognition (Fig. 4B, LS mean difference = -4.04, p = 0.033) and Restricted Interests and Repetitive Behaviour (Fig. 4E, LS mean difference = -3.77, p = 0.037), reflecting improvements in social understanding, cognitive processing, and behavioural flexibility. Positive trends towards improvement were also seen in Social Awareness (Fig. 4A, LS mean difference = -1.89, p = 0.221), and Social Communication (Fig. 4C, LS mean difference = -2.72, p = 0.139). No differences were observed in Social Motivation between groups (Fig. 4D, LS mean difference = -1.47, p = 0.671).

While not statistically significant, the Total T-score for the Active treatment group (NTI164) showed a greater reduction in scores (i.e. improvement) relative to placebo (Fig. 4F, LS mean difference = -2.71, p = 0.055), indicating a positive trend toward improvement in ASD-related social difficulties. See Supplementary Fig. 1 for distribution plots, and Supplementary Table 6 for further descriptive statistics on change from baseline between treatment groups. Collectively, these findings indicate NTI164 can elicit broadly favourable changes in social responsiveness, particularly in domains relating to social cognition and restrictive/repetitive behaviours.

### NTI164 improved anxiety and mood compared to placebo

#### ADAMS

**Fig. 5:**
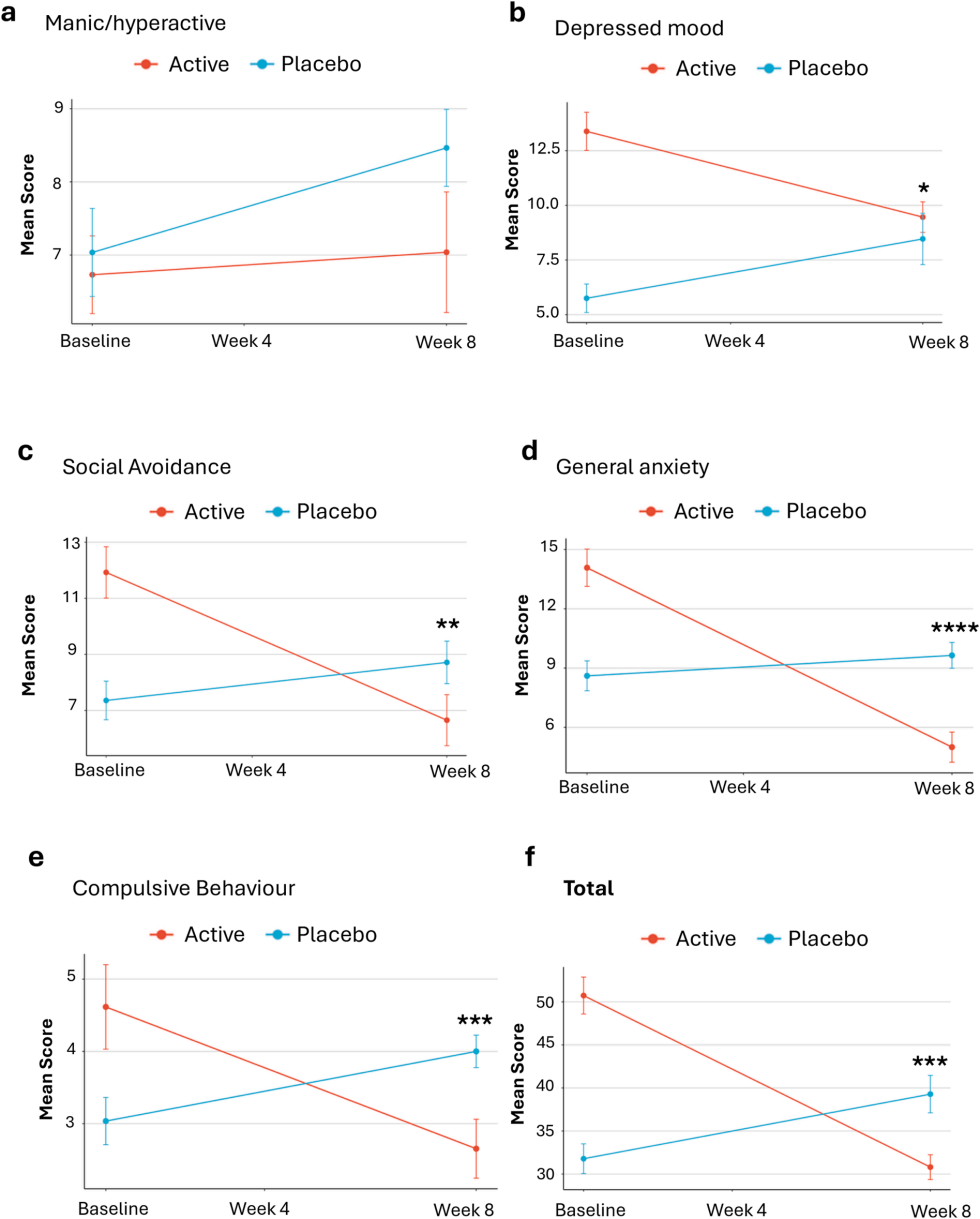
**NTI164 improved anxiety and mood compared to placebo, as measured by the ADAMS** (a) Mean ± change from baseline in Manic/hyperactive scores over time. (b) Mean ± change from baseline in Depressed Mood scores over time. (c) Mean ± change from baseline in Social Avoidance scores over time. (d) Mean ± change from baseline in General Anxiety scores over time. (e) Mean ± change from baseline in Compulsive Behaviour scores over time. (f) Mean ± change from baseline in Total scores over time. n = 54, analysed via ANCOVA, ∗p < 0.05, ∗∗p < 0.01, ∗∗∗p < 0.001, ∗∗∗∗p < 0.0001.

The ADAMS assesses five subdomains: Manic/hyperactive Behaviour, Depressed Mood, Social Avoidance, General Anxiety, and Compulsive Behaviour, with a cumulative Total Score. Assessment of affective and behavioural symptoms using the Anxiety, Depression, and Mood Scale (ADAMS) demonstrated clear and consistent improvements with NTI164 relative to placebo across the 8-week double-blind period (Fig. 5).

Significant treatment effects were observed across multiple subdomains. NTI164 produced significant improvements (i.e. reductions) in Depressed Mood (Fig. 5B, LS mean difference = -4.10, p = 0.019), Social Avoidance (Fig. 5C, LS mean difference = -3.65, p = 0.006), General Anxiety (Fig. 5D, LS mean difference = -5.90, p < 0.0001), and Compulsive Behaviour (Fig. 5E, LS mean difference = -1.64, p = 0.001), reflecting meaningful improvements in emotional distress, internalising symptoms, and maladaptive coping behaviours with NTI164 treatment. Compared to placebo, changes in scores for Manic/hyperactive Behaviour also favoured NTI164, but these were not statistically significant (Fig. 5A, LS mean difference = -1.31, p = 0.162). The LS mean difference (±SE) for the ADAMS Total Score (Fig. 5F) was -18.47 (±3.11), p < 0.001, demonstrating a robust reduction (improvement) in overall affective disturbance in the NTI164 group compared to placebo. See Supplementary Fig. 2 for distribution plots, and Supplementary Table 7 for further descriptive statistics of change from baseline between treatment groups.

#### ASC-ASD-P

**Fig. 6:**
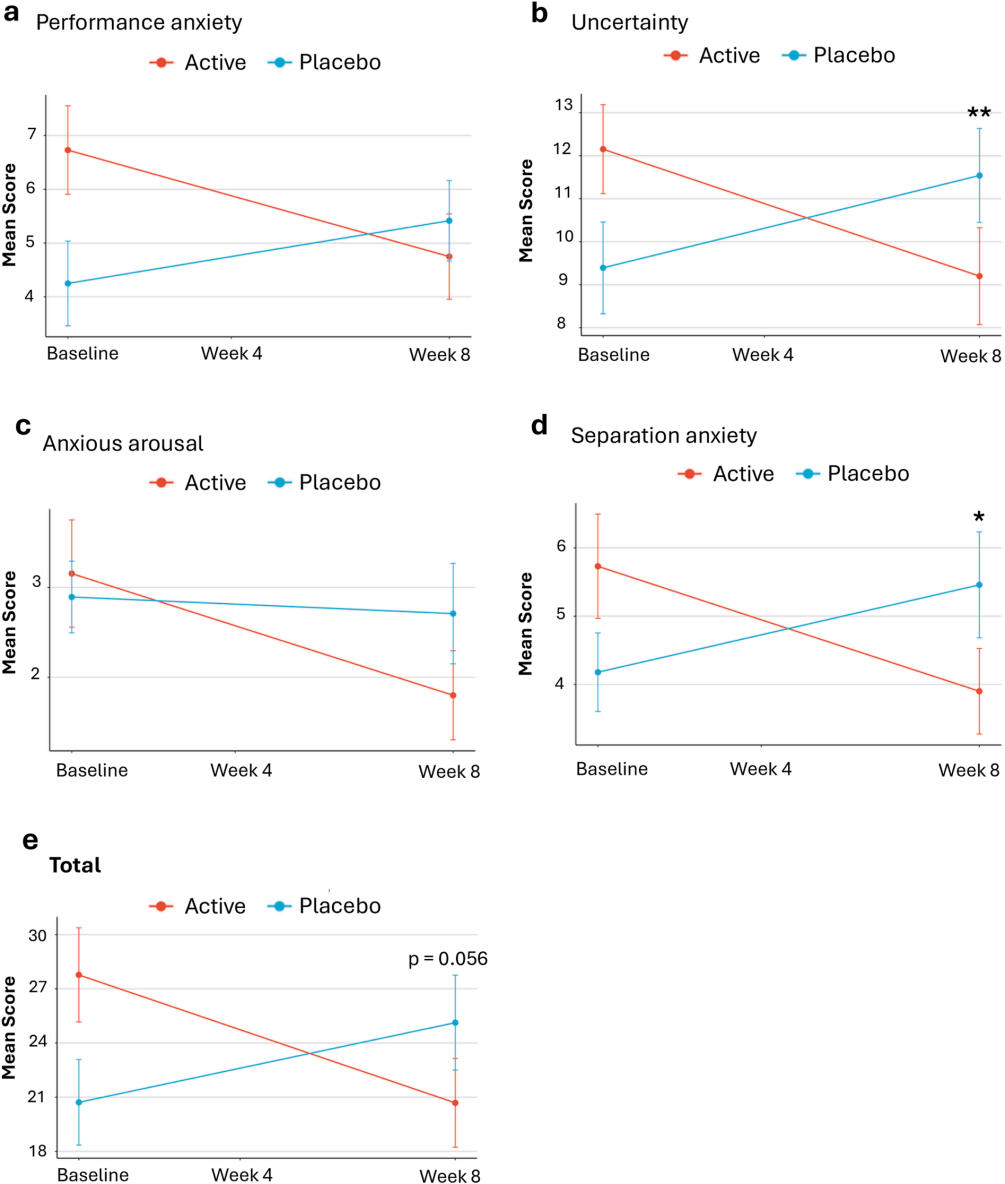
**NTI164 improved anxiety compared to placebo, as measured by the ASC-ASD-P** (a) Mean ± change from baseline in Performance Anxiety scores over time. (b) Mean ± change from baseline in Uncertainty scores over time. (c) Mean ± change from baseline in Anxious Arousal scores over time. (d) Mean ± change from baseline in Separation Anxiety scores over time. (e) Mean ± change from baseline in Total scores over time. n = 54, analysed via ANCOVA, ∗p < 0.05, ∗∗p < 0.01.

The Anxiety Scale for Children-ASD-Parent-rated (ASC-ASD-P) assess symptoms of anxiety over four subdomains: Performance Anxiety, Uncertainty, Anxious Arousal, and Separation Anxiety, with subdomains adding to a Total Score. NTI164 demonstrated favourable improvements over placebo across the 8-week double-blind period (Fig. 6), indicating a clear trend towards reduced overall anxiety in patients receiving NTI164.

Following 8 weeks of oral NTI164 administration, significant improvements were observed in Uncertainty (Fig. 6B, LS mean difference = -4.30, p = 0.008) and Separation Anxiety (Fig. 6D, LS mean difference = -2.43, p = 0.020). Scores in Performance Anxiety (Fig. 6A, LS mean difference = -1.93, p = 0.099) and Anxious Arousal (Fig. 6C, LS mean difference = -1.03, p = 0.231) also decreased in favour of NTI164 but did not reach statistical significance. The LS mean difference (±SE) for the Total Score was -7.85 (±3.99), p = 0.056 (Fig. 6E). See Supplementary Fig. 3 for distribution plots, and Supplementary Table 8 for further descriptive statistics of change from baseline between treatment groups.

### NTI164 improved family experiences compared to placebo

**Fig. 7:**
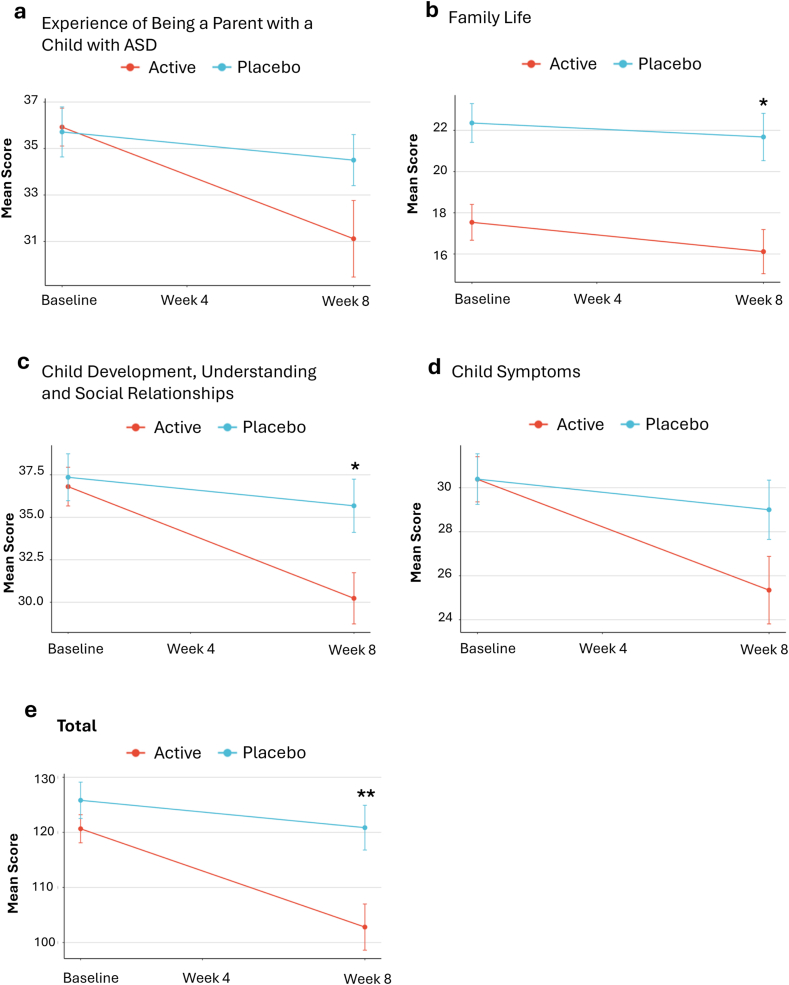
**NTI164 improved family experiences compared to placebo, as measured by the AFEQ** (a) Mean ± change from baseline in Experience of Being a Parent with a Child with ASD scores over time. (b) Mean ± change from baseline in Family Life scores over time. (c) Mean ± change from baseline in Child Development, Understanding and Social Relationships scores over time. (d) Mean ± change from baseline in Child Symptoms scores over time. (e) Mean ± change from baseline in Total scores over time. n = 54, analysed via ANCOVA, ∗p < 0.05, ∗∗p < 0.01.

The Autism Family Experience Questionnaire is a parent/caregiver-rated tool used to measure the impact of autism interventions on family experience and quality of life across four domains: Experience of Being a Parent of a Child with ASD, Family Life, Child Development, Understanding and Social Relationships, and Child Symptoms. Scores across these domains are combined for a Total Score. Assessment with the AFEQ showed significant improvements in family experience and quality of life in the NTI164 group compared to placebo over the 8-week double-blind period (Fig. 7).

Across subdomains, improvements consistently favoured NTI164. Statistically significant improvements (i.e. reductions) were observed in Family Life (Fig. 7B, LS mean difference = -4.33, p = 0.017) and in Child Development, Understanding and Social Relationships (Fig. 7C, LS mean difference = -5.26, p = 0.016). While not reaching statistical significance, directional benefits over placebo were also seen in the Experience of Being a Parent with a Child with ASD (Fig. 7A, LS mean difference -3.44, p = 0.081) and Child Symptoms (Fig. 7D, LS mean difference = -3.65, p = 0.078). The LS mean difference (±SE) for Total Score (Fig. 7E) was -15.73 (±5.66), p = 0.008, indicating a significant reduction over placebo in overall family impact and stress among participants receiving NTI164. See Supplementary Fig. 4 for distributions, and Supplementary Table 9 for further descriptive statistics of change from baseline between treatment groups.

### NTI164 did not improve sleep compared to placebo

**Fig. 8:**
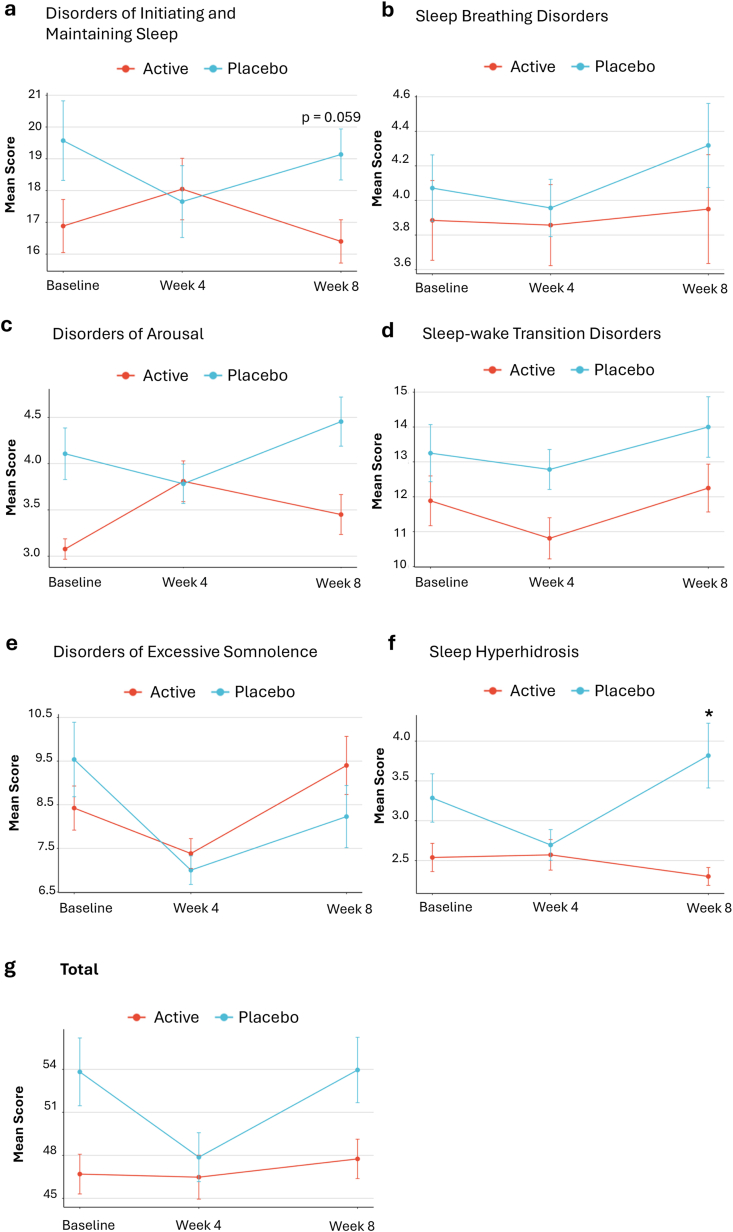
**NTI164 did not improve aspects of sleep compared to placebo, as measured by the SDSC** (a) Mean ± change from baseline in Disorders of Initiating and Maintaining Sleep scores over time. (b) Mean ± change from baseline in Sleep Breathing Disorders scores over time. (c) Mean ± change from baseline in Disorders of Arousal scores over time. (d) Mean ± change from baseline in Sleep-wake Transition Disorders scores over time. (e) Mean ± change from baseline in Disorders of Excessive Somnolence scores over time. (f) Mean ± change from baseline in Sleep Hyperhidrosis scores over time. (g) Mean ± change from baseline in Total scores over time. n = 54, analysed via ANCOVA, ∗p < 0.05.

Sleep outcomes were assessed using the Sleep Disturbances Scale for Children (SDSC) across its Total Score and six subdomains: Disorders of Initiating and Maintaining Sleep (DIMS), Sleep Breathing Disorders (SBD), Disorders of Arousal (DA), Sleep-wake Transition Disorders (SWTD), Disorders of Excessive Somnolence (DOES), and Sleep Hyperhidrosis (SHY). Across the 8-week double-blind period, changes in SDSC scores were generally small in magnitude in both treatment groups (Fig. 8).

Across individual subdomains, trends were variable and did not show clinically meaningful separation between treatment groups (Fig. 8A–E). For DIMS (Fig. 8A), the LS mean difference (±SE) was -2.32 (±1.19), p = 0.059, approached but did not reach statistical significance in favour of NTI164, with both groups showing modest reductions from baseline.

The only subdomain in which a significant difference between treatment groups was observed was SHY (Fig. 8F), where NTI164 was associated with a reduction compared with placebo (LS mean difference = -1.00 (±0.47), p = 0.039). The LS mean difference (±SE) for Total Score was -3.80 (±3.01), p = 0.214, indicating that global sleep disturbance remained largely unchanged over the study period. See Supplementary Fig. 5 for distributions, and Supplementary Table 10 for further descriptive statistics of mean change from baseline.

### NTI164 demonstrated efficacy in core ASD domains in participants transitioning from placebo

Following completion of the 8-week double-blind period, participants originally randomised to the Placebo group were given the opportunity to receive NTI164 open-label if they wished to do so. All participants in the Placebo group elected to try NTI164 open label (n = 28, see Table 2 for age and sex distribution). For these participants, the above efficacy measuring tools were implemented at Week 8 (i.e. prior to beginning NTI164) and at Week 16 (8 weeks following NTI164 introduction). Participants were compared to their own baseline scores, and detailed statistical results are reported in Table 3.

**Table 3:** Descriptive statistics of within-subjects change in all instruments from Week 8 to Week 16 in participants originally allocated to the placebo treatment group following transition to NTI164.

**Parameter**	**n**	**Mean (Week 8)**	**Mean (Week 16)**	**Mean Change**	**SD of Change**	**SE of Change**	**t statistic**	**p value**	**95% CI of Change (low, high**		**Effect Size (paired d)**
**CGI-S**											
Severity of Illness	28	5.25	3.32	−1.93	1.12	0.21	−9.11	<0.001	−2.36	−1.49	−1.72
**CGI-I**											
Total Score	28	4.04	2.25	−1.79	1.60	0.30	−5.92	<0.001	−2.40	−1.17	−1.12
**Vineland-3**											
Adaptive Behaviour Composite	28	51.93	60.93	9.00	5.53	1.05	8.61	<0.001	6.86	11.14	1.63
Communication	28	50.50	54.86	4.36	5.08	0.96	4.54	<0.001	2.39	6.33	0.86
Daily Living Skills	28	52.89	57.11	4.21	4.01	0.76	5.56	<0.001	2.66	5.77	1.05
Socialisation	28	52.54	55.86	3.32	4.88	0.92	3.60	0.001	1.43	5.22	0.68
**SRS-2**											
Total Score	28	77.68	67.86	−9.82	4.53	0.86	−11.47	<0.001	−11.58	−8.06	−2.17
Social Awareness	28	74.89	69.46	−5.43	7.13	1.35	−4.03	<0.001	−8.19	−2.67	−0.76
Social Cognition	28	74.36	69.07	−5.29	5.40	1.02	−5.18	<0.001	−7.38	−3.19	−0.98
Social Communication	28	69.54	66.18	−3.36	3.15	0.60	−5.63	<0.001	−4.58	−2.13	−1.06
Social Motivation	28	67.79	61.79	−6.00	8.18	1.55	−3.88	0.001	−9.17	−2.83	−0.73
Restricted Interest and Repetitive Behaviour	28	70.61	63.89	−6.71	5.91	1.12	−6.01	<0.001	−9.01	−4.42	−1.14
Social Communication and Interaction	28	73.64	67.39	−6.25	7.33	1.39	−4.51	<0.001	−9.09	−3.41	−0.85
**ADAMS**											
Total Score	28	39.29	28.89	−10.39	3.70	0.70	−14.88	<0.001	−11.83	−8.96	−2.81
Manic/Hyperactive Behaviour	28	8.46	6.25	−2.21	0.88	0.17	−13.38	<0.001	−2.55	−1.87	−2.53
Depressed Mood	28	8.46	6.21	−2.25	1.71	0.32	−6.95	<0.001	−2.91	−1.59	−1.31
Social Avoidance	28	8.71	6.43	−2.29	1.21	0.23	−9.97	<0.001	−2.76	−1.82	−1.88
General Anxiety	28	9.64	7.04	−2.61	1.07	0.20	−12.94	<0.001	−3.02	−2.19	−2.45
Compulsive Behaviour	28	4.00	2.96	−1.04	0.33	0.06	−16.54	<0.001	−1.16	−0.91	−3.13
**AFEQ**											
Total Score	28	117.64	113.54	−4.11	7.34	1.39	−2.96	0.006	−6.95	−1.26	−0.56
Experience of Being a Parent of a Child with Autism	27	33.78	32.48	−1.30	2.22	0.43	−3.04	0.005	−2.17	−0.42	−0.59
Family Life	28	21.68	20.68	−1.00	1.19	0.22	−4.46	<0.001	−1.46	−0.54	−0.84
Child Development, Understanding and Social Relationships	28	35.68	33.75	−1.93	1.54	0.29	−6.64	<0.001	−2.52	−1.33	−1.25
Child Symptoms	28	29.00	27.79	−1.21	1.52	0.29	−4.22	<0.001	−1.81	−0.62	−0.80
**SDSC**											
Total Score	15	53.13	52.87	−0.27	8.06	2.08	−0.13	0.900	−4.73	4.20	−0.03
Disorder of Initiating and Maintaining Sleep	15	19.47	19.07	−0.40	4.70	1.21	−0.33	0.747	−3.00	2.20	−0.09
Sleep Breathing Disorders	15	3.87	4.07	0.20	1.61	0.42	0.48	0.638	−0.69	1.09	0.12
Disorders of Arousal	15	4.80	3.40	−1.40	1.64	0.42	−3.31	0.005	−2.31	−0.49	−0.85
Sleep-wake transition disorders	15	14.07	13.73	−0.33	4.05	1.05	−0.32	0.754	−2.57	1.91	−0.08
Disorders of excessive somnolence	15	7.60	10.13	2.53	4.34	1.12	2.26	0.040	0.13	4.94	0.58
Sleep hyperhidrosis	15	3.33	2.47	−0.87	1.51	0.39	−2.23	0.043	−1.70	−0.03	−0.58
**ASC-ASD-P**											
Total Score	16	25.19	19.88	−5.31	7.34	1.83	−2.90	0.011	−9.22	−1.40	−0.72
Performance Anxiety	17	4.47	3.47	−1.00	2.81	0.68	−1.47	0.161	−2.44	0.44	−0.36
Anxious Arousal	17	2.47	2.35	−0.12	1.73	0.42	−0.28	0.783	−1.01	0.77	−0.07
Separation Anxiety	17	5.53	4.71	−0.82	2.35	0.57	−1.44	0.168	−2.03	0.39	−0.35
Uncertainty	17	11.65	8.18	−3.47	3.94	0.96	−3.63	0.002	−5.50	−1.45	−0.88

Within-subject comparisons demonstrated consistent improvements across clinician- and caregiver-rated outcomes after participants transitioned from placebo to NTI164. Clinician-rated global measures showed clear improvement following NTI164 treatment, with CGI-S scores decreasing by a mean of -1.93 points (p < 0.001) and CGI-I scores improving by a mean -1.79 points (p < 0.001). These findings support meaningful overall clinical benefit.

Adaptive functioning, assessed using the Vineland-3, showed marked gains across all domains. Significant improvements were observed in the ABC (+9.0, p < 0.001), Communication (+4.36, p < 0.001), Daily Living Skills (+4.21, p < 0.001), and Socialisation (+3.32, p = 0.001), reflecting broad positive shifts in day-to-day function in participants upon receiving NTI164.

The SRS-2 also revealed improvements in social responsiveness in participants treated with NTI164, with reductions in Total T-score (-9.82, p < 0.001) and in multiple subdomains, including Social Awareness, Social Cognition, Social Communication, Social Motivation, and Restricted Interests and Repetitive Behaviour.

Affective symptoms assessed by the ADAMS demonstrated robust reductions following NTI164 initiation. Total Score improved by -10.39 (p < 0.001), alongside significant decreases in Manic/hyperactive Behaviour, Depressed Mood, Social Avoidance, General Anxiety, and Compulsive Behaviour (all p < 0.001), showing broad reductions in emotional and behavioural distress. Anxiety symptoms on the ASC-ASD-P also demonstrated reductions during NTI164 treatment, with significant improvements in Total Score (-5.31; p = 0.011) and in several subdomains, including Separation Anxiety (-0.82; p = 0.037) and Uncertainty (-3.47; p = 0.002), highlighting enhanced emotional regulation and reduced worry.

Family-reported outcomes using the AFEQ showed favourable within-subject improvement, including reductions in Total Score (-4.11; p = 0.006) and consistent positive changes across multiple domains related to family life, parenting experience, child behaviour, and social understanding.

Sleep outcomes assessed via the SDSC were largely stable, with a modest improvement in Disorders of Excessive Somnolence (+2.53; p = 0.040) and small directional changes across other subdomains, consistent with the results seen in the original double-blind study period. Collectively, these exploratory within-subject findings indicate that participants who initially received placebo exhibited consistent and clinically relevant improvements across functional, behavioural, emotional, and family-reported measures once NTI164 treatment commenced.

## Discussion

In this randomised, double-blind, placebo-controlled trial, NTI164 demonstrated statistically significant efficacy across clinician-rated, caregiver-rated, and functional outcome measures in children and adolescents with Level II or III (moderate-severe) ASD. Participants who received NTI164 showed significant improvements in the primary outcome, overall clinical severity (CGI-S; Fig. 1), as well as secondary outcomes including global impression of clinical improvement (CGI-I; Fig. 2), adaptive functioning (Vineland-3; Fig. 3), social responsiveness (SRS-2; Fig. 4) affective symptoms (ADAMS; Fig. 5), anxiety (ASC-ASD-P; Fig. 6), and family quality of life (AFEQ; Fig. 7), compared with placebo over the 8-week double-blind main study period. Improvements were multi-dimensional, and included core ASD symptoms, behavioural symptoms, and family impact.

NTI164 was determined to be safe and well-tolerated in this paediatric cohort of ASD patients. All reported AEs were deemed mild, transient, and non-serious by study clinicians, and no serious AEs were reported during the study period. AEs were comparable across treatment groups, suggesting they could be due to the olive oil vehicle rather than actives of NTI164. No significant clinical laboratory or vital sign abnormalities were reported throughout the study period. Additionally, the high completion rate (∼88%), as well as low discontinuation due to AEs, supports the tolerability of NTI164 and further clinical development in this patient population.

Current available treatments for ASD primarily target irritability or related behavioural symptoms, rather than core ASD symptoms. A difference of 1.4 on the CGI-I scale for participants receiving NTI164 (Fig. 2) indicates, on average, participants in the Active group were rated as improved relative to their baseline regarding their overall clinical presentation, compared to the Placebo group who remained close to “no change”. Given the broad and often persistent nature of ASD symptoms, this magnitude of improvement is considered clinically meaningful and suggests noticeable benefit across multiple domains of functioning. The large standard deviation observed in the placebo group suggests greater variability in individual responses compared with the active group (Fig. 2). This may reflect the heterogenous nature of placebo responses commonly observed in ASD clinical trials, where participants can exhibit varying degrees of perceived improvement due to factors such as natural symptom fluctuation, caregiver expectations, and environmental influences [27]. In contrast, the lower variability in responses observed in the NTI164 group may indicate a more consistent treatment effect across participants, however, warrants confirmation in larger studies.

The core symptoms of ASD are widely regarded as relating to social communication/interaction and restricted, repetitive behaviours or interests. Thus, adaptive functioning and social responsiveness represent arguably the most clinically meaningful outcomes in ASD, reflecting improvements in real-world daily capabilities. In this study, 8 weeks of oral treatment with NTI164 at doses of 10–20 mg/kg/day produced statistically significant improvements across all domains in the Vineland-3, including the ABC (Fig. 3). These findings suggest improvements seen with NTI164 extend beyond symptom reduction to meaningful improvements in day-to-day functioning in domains relating to communication, daily living skills, and socialisation. Consistent with the improvements seen using the Vineland-3, the SRS-2 also demonstrated significant improvements across domains of social responsiveness, particularly in Social Cognition (Fig. 4B) and Restricted Interests and Repetitive Behaviour (Fig. 4E). While the Total T-score narrowly remained above the limit of statistical significance for the double-blind phase of this study (Fig. 4f, p = 0.055), the directionality of effect, as well as the reported improvements seen in children following transition from placebo to open-label use of NTI164, suggest the potential for a clinically meaningful effect that warrants further investigation in larger studies. The congruence between these clinician- and caregiver-rated tools strengthens the claim that observed benefits reflect tangible, real clinical benefit.

Emotional dysregulation and anxiety are also highly prevalent in ASD and can contribute immensely to impairment. In the Harmony study, the ADAMS assessment showed NTI164 elicited robust reductions (i.e. improvements) in affective disturbances across all subdomains, with the only exception of Manic/hyperactive Behaviour (Fig. 5). The substantial improvement in the ADAMS Total Score in patients receiving NTI164 compared to placebo (p < 0.001) suggests clinically meaningful improvements in the burden of emotional symptoms. Similarly, the ASC-ASD-P showed improved emotional regulation and reduced anxiety, particularly in the domains of Uncertainty (Fig. 6B) and Separation Anxiety (Fig. 6D).

In addition to patients, ASD places a significant toll on families as well. NTI164 demonstrated significant improvements over placebo in family experience and quality of life, as measured by the AFEQ (Fig. 7). Benefits were particularly pronounced in the Family Life (Fig. 7B) and Child Development, Understanding and Social Relationships (Fig. 7C) subdomains, underscoring the broader psychosocial impact treatment with NTI164 may have. An improvement in family experiences could in turn lead to improved household stability, reduced stress, and overall enhanced family wellbeing.

Sleep disturbance scores did not significantly differ across treatment groups over the 8-week double-blind study period, with the exception of a modest improvement in Sleep Hyperhidrosis for patients receiving NTI164 (Fig. 8F). Largely though, sleep measures remained unchanged, suggesting the observed clinical benefits identified through other assessment tools were not secondary to improved sleep and may instead reflect direct effects on mechanisms underlying ASD symptom development and maintenance.

Similarly to the initial 8-week double-blind phase of the study, participants originally randomised to placebo who then transitioned to open-label use of NTI164 demonstrated consistent reported improvements across clinician- and caregiver-rated domains, as well as functional outcomes. Within-subject improvements reported with the CGI scales, Vineland-3, SRS-2, ADAMS, ASC-ASD-P, and AFEQ reinforce the reproducibility and durability of the observed treatment effects. Although open-label findings require more cautious interpretation due to the absence of blinding, the consistency of these findings across many independent measures lends strength to the overall suggested efficacy of NTI164 in an ASD population.

The pattern of improvement observed in the current study with NTI164 treatment across areas of social cognition and responsiveness, emotional regulation, anxiety, and behavioural flexibility is consistent with modulation of neural systems implicated in ASD. Cannabinoids exert a variety of effects on neuroinflammatory pathways, excitatory/inhibitory neurotransmitter balance, and stress response pathways through modulation of the endocannabinoid system (ECS) [23,28], with cannabinoid receptors widely expressed in neurons [29], microglia, and astrocytes [30]. Activation of these receptors, both CB1 and CB2, has been shown to suppress neuroinflammation by reducing release of pro-inflammatory cytokines, ameliorating microglial activation [29], and maintaining blood-brain-barrier integrity [31], in turn limiting neuronal damage and dysfunction. These anti-inflammatory benefits are particularly relevant in ASD, where elevated inflammatory markers and immune dysregulation have been consistently reported [1].

Cannabinoids also influence neurotransmitter balance by modulating presynaptic neurotransmitter release [32,33]. Activation of CB1 receptors has been reported to suppress excessive glutamatergic excitation and supporting GABA levels [34], thereby stabilising neuronal function and reducing damage via excitotoxicity [35], which has been implicated in ASD impairments relating to cognition and social behaviour [36]. In parallel, the ECS regulates the hypothalamic-pituitary-adrenal (HPA) axis and stress responsivity, promoting adaptive recovery in stressful environments [37,38]. Dysregulation of stress responses is prevalent in ASD, contributing to symptoms of anxiety, behavioural rigidity and reactivity, and emotional dysregulation. Through synergistic regulation of immune signalling, neurotransmitter homeostasis, and regulating stress responses, cannabinoids address fundamental biological mechanisms underpinning core and associated ASD symptoms [39].

The findings of the Harmony study are consistent with a growing body of evidence supporting the therapeutic role of cannabinoids in ASD and other neurodevelopmental disorders. CBD isolates and CBD-rich formulations have reported improvements in behavioural symptoms of ASD [40]. In addition to CBD, NTI164 contains predominantly CBDA, as well as other minor cannabinoids, including extremely low levels of THC and THCA. Although clinical data for CBDA and THCA in ASD remain limited, these cannabinoids have been shown to influence biological pathways implicated in neurodevelopmental disorders, such as neuroinflammation, serotonergic signalling, and ECS regulation [[41], [42], [43]]. The results reported in our current study extend previous findings with cannabinoid-based therapies, particularly multi-constituent formulations such as NTI164.

Evidence suggests NTI164 exerts disease modifying effects in neurodevelopmental disorders through its anti-inflammatory [24] and epigenetic regulatory properties, which may directly target pathophysiological mechanisms in ASD. In a clinical trial of paediatric acute-onset neuropsychiatric syndrome (PANS), in which more than half of participants had a comorbid ASD diagnosis, multi-omic analyses showed NTI164 significantly modulated dysregulated immune and epigenetic cellular pathways, normalising aberrant gene expression pathways associated with neurodevelopmental dysfunction [44]. These epigenetic effects are particularly relevant in ASD as there is increasing recognition of altered gene expression and neuroimmune interactions affecting synaptic and neuronal health in this patient population [6,15,45].

Clinically, NTI164 has repeatedly demonstrated improvements in emotional regulation, anxiety, obsessive/compulsive behaviour, autonomic function, and quality of life in paediatric cohorts of neurodevelopmental disorders, alongside reductions in scores of clinical severity [26,44,46]. Notably, previous clinical trials have demonstrated improvements in behaviour and affective symptoms extending beyond the patient to the wider family unit, improving family dynamics and caregiver burden [46]. The benefits reported in the Harmony study support the hypothesis that NTI164's anti-inflammatory and epigenetic mechanisms may enhance neuronal health, and improve symptoms of ASD. Collectively, these data suggest NTI164 may act as a disease-modifying therapeutic improving biological processes underlying core ASD symptoms, rather than providing purely symptomatic relief.

The randomised, double-blind, placebo-controlled design of the Harmony study allowed data to be generated in a scientifically rigorous and valid manner uncommon in the field of cannabis in ASD. The consistency in findings and use of validated clinician-rated questionnaires, administered by trained personnel, as well as caregiver-rated instruments also supports the weight of evidence that NTI164 is safe and efficacious in paediatric ASD cohorts. However, several limitations of the current study remain, including a relatively short double-blind period of 8 weeks, and the small sample size and single site for a condition as widespread and diverse as ASD. The assessment of outcomes following 8 weeks of NTI164, and the allowance of the maximum tolerated dose to be patient-specific (e.g. target dose of 20 mg/kg/day or less if the patient could not tolerate the target dose), allowed a heterogeneous exposure period. Consequently, efficacy assessments at Week 8 may reflect differing durations of exposure at therapeutic dose levels, potentially contributing to variability in treatment response. A multi-centre Phase III randomised, double-blind, placebo-controlled clinical trial of NTI164 in ASD is currently in development (NTIASD3, ClinicalTrial.gov ID: NCT07257939) and will involve a larger patient population defined by formal power calculations, as well as exploratory multi-omics analyses, including transcriptomics and proteomics, to further investigate the mechanism of action of NTI164 in ASD. The treatment period (i.e. post up-titration) will be 12 weeks in this study, as opposed to 8, and patients’ maximum tolerated doses will be factored into statistical analyses. As well, significant impacts of placebo effects of caregivers of children with ASD have been reported in previous studies [27]. The current study did not assess this, and future studies may benefit from capturing this information, potentially through asking caregivers prior to unblinding which treatment arm they believe they were in.

The multi-domain improvements observed with NTI164 treatment compared to placebo reported in this study support utility as a broader therapeutic intervention which can address ASD symptoms. This randomised, double-blind, placebo-controlled clinical trial in children and adolescents with moderate-severe ASD showed NTI164 meaningfully improves global impression of clinical severity, adaptive functioning, aspects of social responsiveness, mood, anxiety, and family experiences, with an excellent safety profile. The reproducibility of benefits across clinician- and caregiver-rated tools supports further confirmatory investigation of NTI164 as a novel therapeutic in ASD.

## Product availability

NTI164 is currently unregistered and can only be accessed through clinical trials, or compassionate use programs at the discretion of Authorised Prescribers. It is currently not available in any capacity outside of Australia.

## Data availability

Additional data not presented here is available upon request by qualified Investigators. Requests for further information should be directed to the corresponding author.

## Author contributions

YO, EI, DE, and MCF designed the trial. KS, DE, and MCF managed and recruited patients, and performed clinical assessments. BAK and YO oversaw data presentation. BAK and YO drafted this manuscript. All authors critically revised this manuscript.

## Funding statement

This study was monetarily supported by Fenix Innovation Group Pty Ltd and Neurotech International Ltd (Sponsors). The Sponsors had no role in data collection or analysis.

## Declaration of competing interest

The authors declare the following financial interests/personal relationships which may be considered as potential competing interests:

Brooke A Keating reports financial support was provided by Fenix Innovation Group. Yelda Ogru reports financial support was provided by Fenix Innovation Group. Esra Isikgel reports financial support was provided by Fenix Innovation Group. Michael C Fahey reports a relationship with Neurotech International Ltd that includes: consulting or advisory. Esra Isikgel reports a relationship with Neurotech International Ltd that includes: equity or stocks. No other interests to declare. If there are other authors, they declare that they have no known competing financial interests or personal relationships that could have appeared to influence the work reported in this paper.
