# Supplementary material for: NTI164, a novel medicinal cannabis extract, improves core symptoms of autism spectrum disorder: Results from a double-blind, randomised, controlled trial (The Harmony study)
Source: Neurotherapeutics. 2026 Aug 6;23(5):e01033. doi: 10.1016/j.neurot.2026.e01033 (PMC13474237; doi:10.1016/j.neurot.2026.e01033)
Supplement: Supplementary file 1 [file mmc1.pdf]

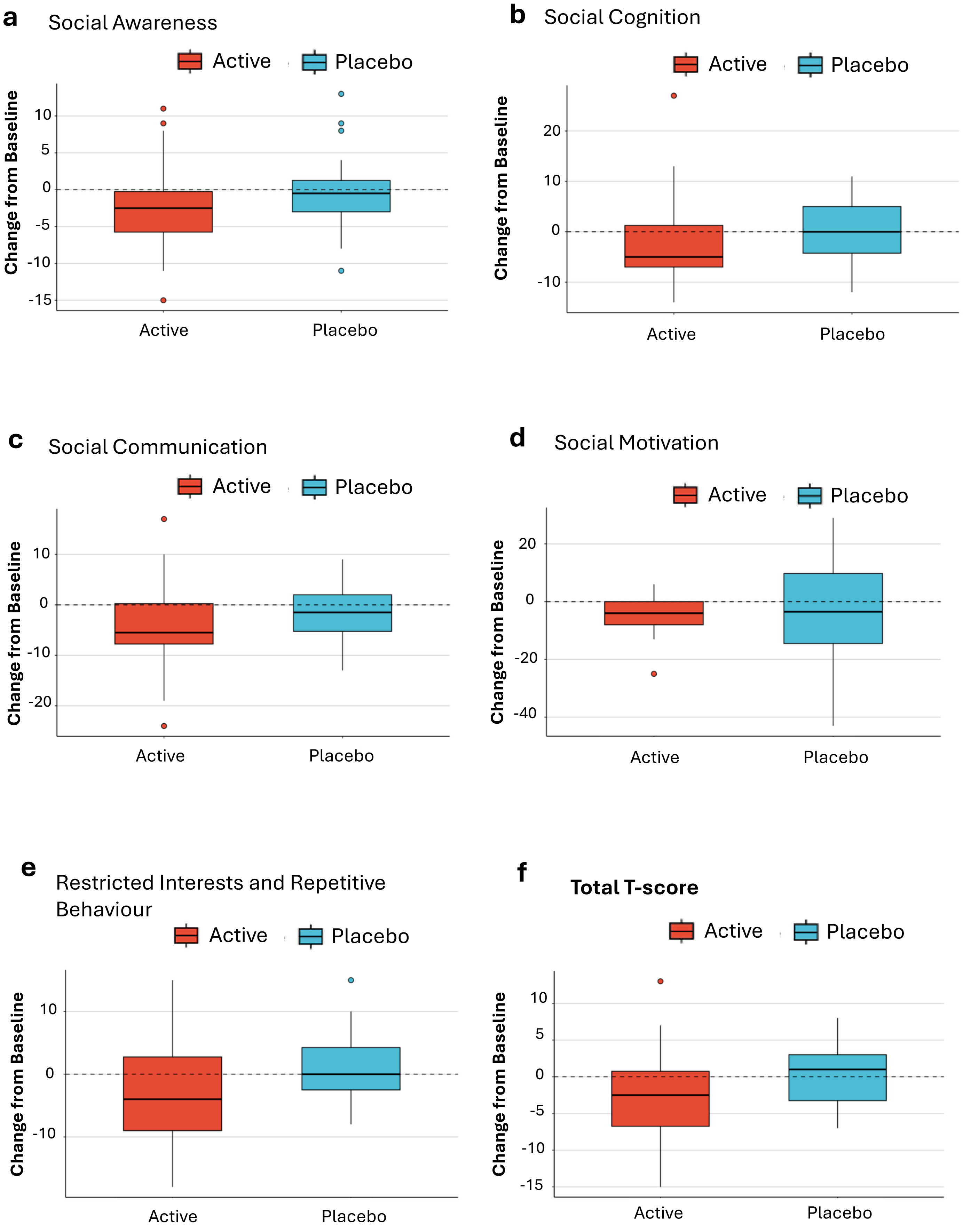
a
Social Awareness
Active
Placebo
Change from Baseline
10
5
0
-5
-10
-15
Active
Placebo
b
Social Cognition
Active
Placebo
Change from Baseline
20
10
0
-10
Active
Placebo
c
Social Communication
Active
Placebo
Change from Baseline
10
0
-10
-20
Active
Placebo
d
Social Motivation
Active
Placebo
Change from Baseline
20
0
-20
-40
Active
Placebo
e
Restricted Interests and Repetitive Behaviour
Active
Placebo
Change from Baseline
10
0
-10
Active
Placebo
f
Total T-score
Active
Placebo
Change from Baseline
10
5
0
-5
-10
-15
Active
Placebo
