# Supplementary material for: NTI164, a novel medicinal cannabis extract, improves core symptoms of autism spectrum disorder: Results from a double-blind, randomised, controlled trial (The Harmony study)
Source: Neurotherapeutics. 2026 Aug 6;23(5):e01033. doi: 10.1016/j.neurot.2026.e01033 (PMC13474237; doi:10.1016/j.neurot.2026.e01033)
Supplement: Supplementary file 2 [file mmc2.pdf]

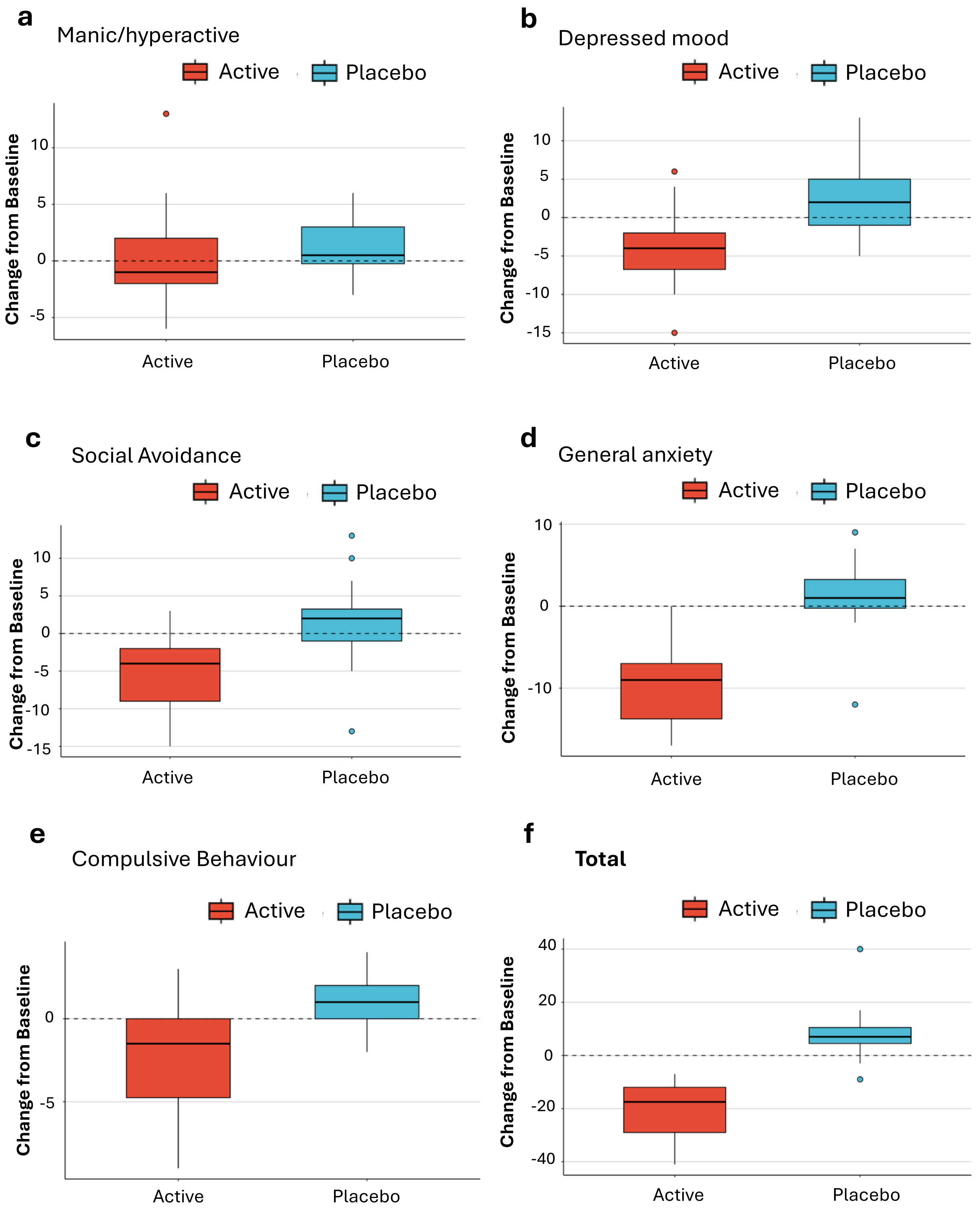
a
Manic/hyperactive
Active
Placebo
Change from Baseline
10
5
0
-5
Active
Placebo
b
Depressed mood
Active
Placebo
Change from Baseline
10
5
0
-5
-10
-15
Active
Placebo
c
Social Avoidance
Active
Placebo
Change from Baseline
10
5
0
-5
-10
-15
Active
Placebo
d
General anxiety
Active
Placebo
Change from Baseline
10
0
-10
Active
Placebo
e
Compulsive Behaviour
Active
Placebo
Change from Baseline
0
-5
Active
Placebo
f
Total
Active
Placebo
Change from Baseline
40
20
0
-20
-40
Active
Placebo
