# Supplementary material for: NTI164, a novel medicinal cannabis extract, improves core symptoms of autism spectrum disorder: Results from a double-blind, randomised, controlled trial (The Harmony study)
Source: Neurotherapeutics. 2026 Aug 6;23(5):e01033. doi: 10.1016/j.neurot.2026.e01033 (PMC13474237; doi:10.1016/j.neurot.2026.e01033)
Supplement: Supplementary file 3 [file mmc3.pdf]

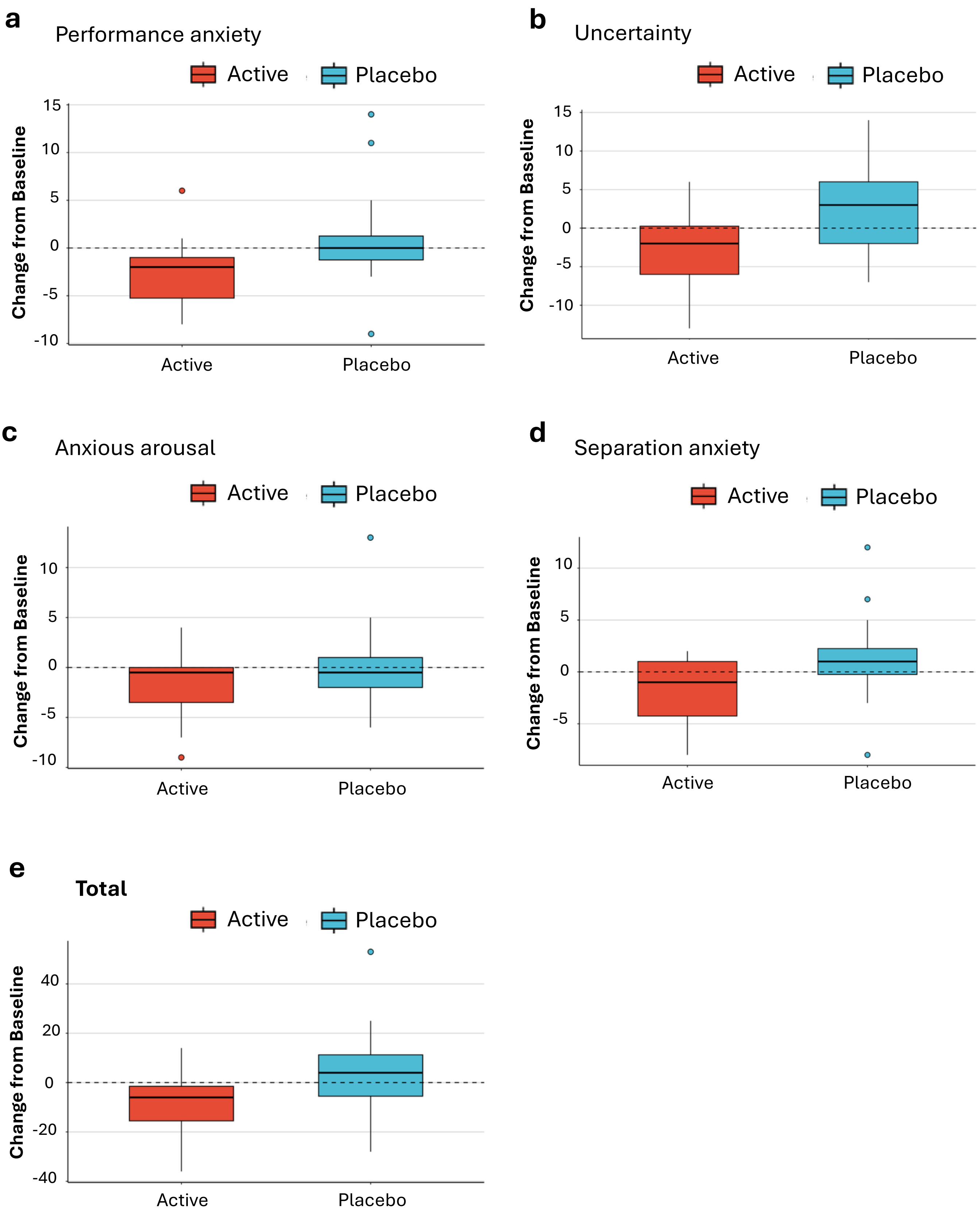

a
Performance anxiety
Active
Placebo
Change from Baseline
15
10
5
0
-5
-10
Active
Placebo
b
Uncertainty
Active
Placebo
Change from Baseline
15
10
5
0
-5
-10
Active
Placebo
c
Anxious arousal
Active
Placebo
Change from Baseline
10
5
0
-5
-10
Active
Placebo
d
Separation anxiety
Active
Placebo
Change from Baseline
10
5
0
-5
Active
Placebo
e
Total
Active
Placebo
Change from Baseline
40
20
0
-20
-40
Active
Placebo
