# Supplementary material for: NTI164, a novel medicinal cannabis extract, improves core symptoms of autism spectrum disorder: Results from a double-blind, randomised, controlled trial (The Harmony study)
Source: Neurotherapeutics. 2026 Aug 6;23(5):e01033. doi: 10.1016/j.neurot.2026.e01033 (PMC13474237; doi:10.1016/j.neurot.2026.e01033)
Supplement: Supplementary file 4 [file mmc4.pdf]

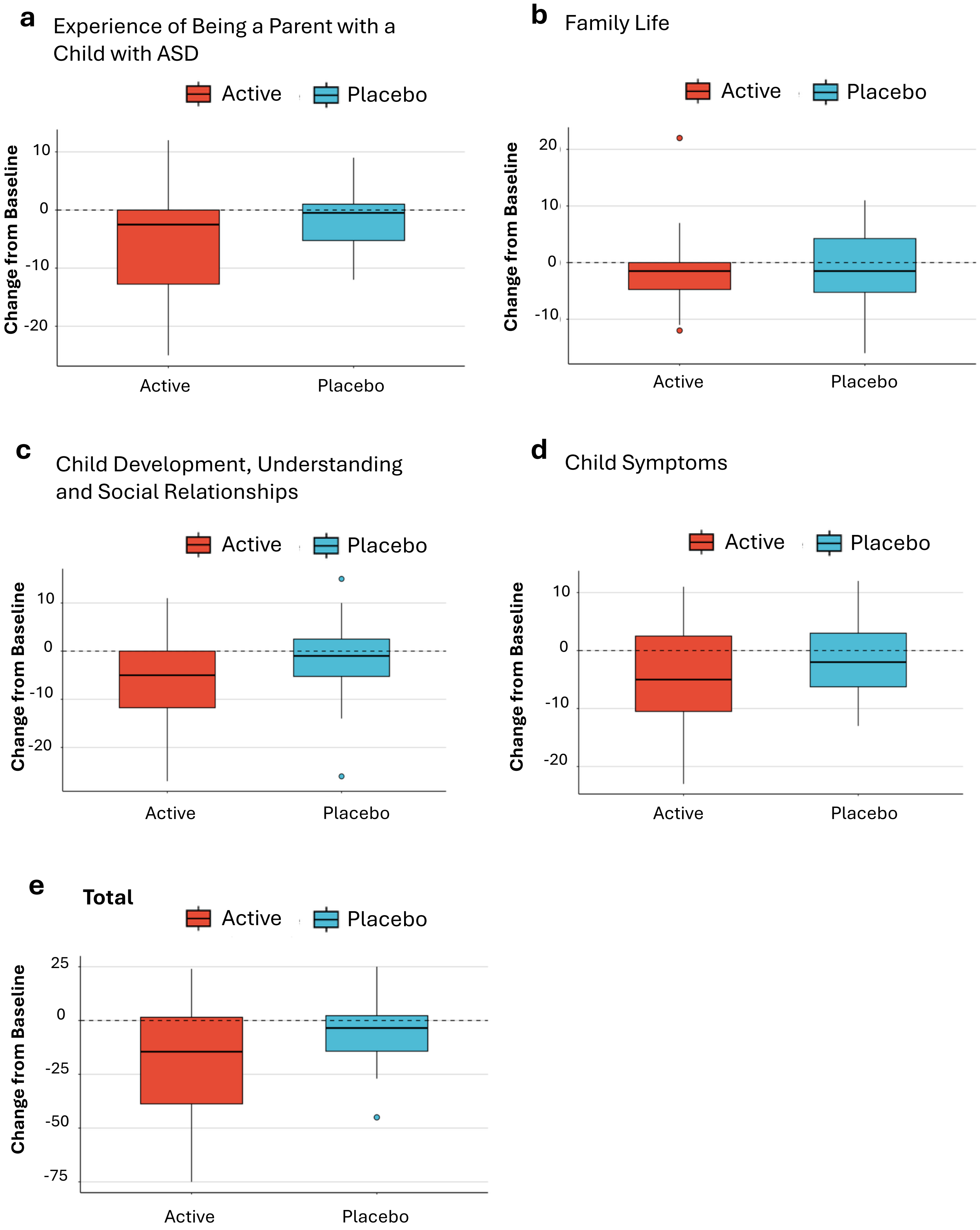

a
Experience of Being a Parent with a Child with ASD
Active
Placebo
Change from Baseline
10
0
-10
-20
Active
Placebo
b
Family Life
Active
Placebo
Change from Baseline
20
10
0
-10
Active
Placebo
c
Child Development, Understanding and Social Relationships
Active
Placebo
Change from Baseline
10
0
-10
-20
Active
Placebo
d
Child Symptoms
Active
Placebo
Change from Baseline
10
0
-10
-20
Active
Placebo
e
Total
Active
Placebo
Change from Baseline
25
0
-25
-50
-75
Active
Placebo
