# Supplementary material for: NTI164, a novel medicinal cannabis extract, improves core symptoms of autism spectrum disorder: Results from a double-blind, randomised, controlled trial (The Harmony study)
Source: Neurotherapeutics. 2026 Aug 6;23(5):e01033. doi: 10.1016/j.neurot.2026.e01033 (PMC13474237; doi:10.1016/j.neurot.2026.e01033)
Supplement: Supplementary file 5 [file mmc5.pdf]

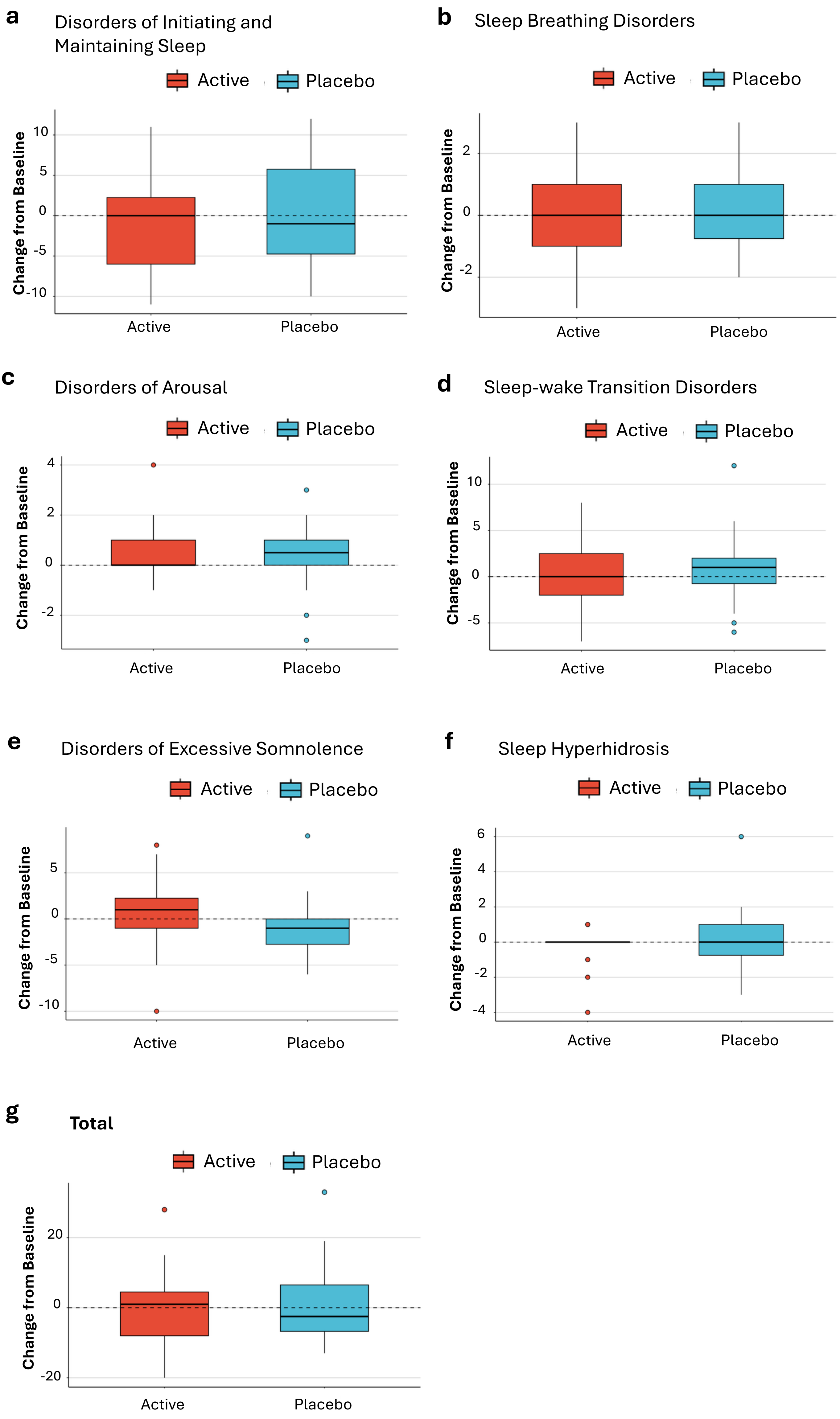
a
Disorders of Initiating and Maintaining Sleep
Active
Placebo
Change from Baseline
10
5
0
-5
-10
Active
Placebo
b
Sleep Breathing Disorders
Active
Placebo
Change from Baseline
2
0
-2
Active
Placebo
c
Disorders of Arousal
Active
Placebo
Change from Baseline
4
2
0
-2
Active
Placebo
d
Sleep-wake Transition Disorders
Active
Placebo
Change from Baseline
10
5
0
-5
Active
Placebo
e
Disorders of Excessive Somnolence
Active
Placebo
Change from Baseline
5
0
-5
-10
Active
Placebo
f
Sleep Hyperhidrosis
Active
Placebo
Change from Baseline
6
4
2
0
-2
-4
Active
Placebo
g
Total
Active
Placebo
Change from Baseline
20
0
-20
Active
Placebo
