# Supplementary material for: NTI164, a novel medicinal cannabis extract, improves core symptoms of autism spectrum disorder: Results from a double-blind, randomised, controlled trial (The Harmony study)
Source: Neurotherapeutics. 2026 Aug 6;23(5):e01033. doi: 10.1016/j.neurot.2026.e01033 (PMC13474237; doi:10.1016/j.neurot.2026.e01033)
Supplement: Supplementary file 6 [file mmc6.docx]

**SUPPLEMENTARY MATERIAL**

**Supplementary Material, Appendix 1. Inclusion/exclusion criteria for NTIASD2 (Harmony) study.**

*Inclusion*

- Participant is 8-17 years of age at the time of enrolment (inclusive).
- Participant is at a healthy weight, as determined by the Principal Investigator.
- Participant and consent-giver can comply with trial requirements.
- Participant has a diagnosis of Level II or Level III Autism Spectrum Disorder (ASD) as defined by the Diagnostic and Statistical Manual of Mental Disorders, 5^th^ Edition (DSM-5), confirmed by a validated assessment tool.
- All other treatments for ASD-related symptoms (including medications and other therapies) must have been stable for at least 4 weeks prior to enrolment and for the duration of the trial.
- Participant must be able to swallow liquid.
- Written informed consent is obtained from parent/s or legal guardian, according to the local law.

*Exclusion*

- Current diagnosis of bipolar disorder, psychosis, schizophrenia, schizoaffective disorder, or active major depression.
- Has a diagnosis other than ASD which dominates the clinical presentation (e.g. attention deficit hyperactivity disorder (ADHD)), as determined by the Principal Investigator.
- Has a degenerative condition.
- Participant has had anti-convulsive medication changed or dose adjusted in the 12 weeks prior to enrolment.
- Taking omeprazole, lansoprazole, tolbutamide, warfarin, sirolimus, everolimus, temsirolimus, tacrolimus, clobazam, repaglinide, pioglitazone, rosiglitazone, montelukast, bupropion, or efavirenz.
- Currently using or has used recreational or medicinal cannabis or cannabinoid-based medications (including Sativex® or Epidiolex®) within the 12 weeks prior to screening and is unwilling or unable to abstain for the duration of the trial.
- Participant has any known or suspected sensitivity to cannabinoids or any of the excipients in NTI164.
- Participant has moderately (or worse) impaired hepatic function at screening, defined as serum alanine transferase (ALT) or aspartate aminotransferase (AST) > 2 x upper limit of normal (ULN) or total bilirubin (TBL) > 2 x ULN. This criterion can only be confirmed once laboratory results are available, participants enrolled into the trial later found to meet this criterion will be screen-failed.

**Supplementary Table 1. Clinical pathology parameters for safety monitoring in the NTIASD2 clinical trial.**

| Haematology | Haematocrit, haemoglobin, mean cell haemoglobin (MCH), mean cell volume (MCV), mean corpuscular haemoglobin concentration (MCHC), platelets, mean platelet volume (MPV), red blood cell (RBC) count, RBC distribution width, white cell count, basophils, eosinophils, lymphocytes, monocytes, neutrophils |
| --- | --- |
| Clinical chemistry | Albumin, alkaline phosphatase (ALP), alanine transaminase (ALT), anion gap, aspartate transferase (AST), bicarbonate, bilirubin (total, direct, & indirect), calcium (adjusted), calcium, chloride, creatinine, estimated glomerular filtration rate (eGFR), gamma-glutamyl transferase (GGT), globulin, lactate dehydrogenase (LD), phosphorus, potassium, protein, sodium, urea, uric acid |
| Coagulation | International normalised ratio (INR), prothrombin time, activated partial thromboplastin time (aPTT) |

**Supplementary Table 2. Adverse events reported during the NTIASD2 clinical trial.**

| System Organ Class  Preferred Term | NTI164  (n = 26) | Placebo  (n = 28) | Total  (n = 54) |
| --- | --- | --- | --- |
| **Gastrointestinal disorders** |  |  |  |
| Abdominal pain | 4 | 3 | 7 |
| Nausea | 4 | 5 | 9 |
| Diarrhoea | 0 | 1 | 1 |
| **Nervous system disorders** |  |  |  |
| Headache | 3 | 5 | 8 |
| **Psychiatric disorders** |  |  |  |
| Anxiety | 3 | 3 | 6 |
| Agitation | 0 | 3 | 3 |
| **Respiratory, thoracic and mediastinal disorders** |  |  |  |
| Cough | 1 | 2 | 3 |
| Sore throat | 0 | 1 | 1 |
| **General disorders and administration-site conditions** |  |  |  |
| Pyrexia | 0 | 2 | 2 |
| **Metabolism and nutrition disorders / Other** |  |  |  |
| Lethargy | 0 | 1 | 1 |
| **Total AEs** | **15** | **26** | **41** |
| **Total Patients** | **13** | **13** | **26** |

**Supplementary Table 3. Change in baseline in CGI-S per treatment group.**

| Group | N | Baseline Mean Score | Week 8 Mean Score | Mean Change | SD |
| --- | --- | --- | --- | --- | --- |
| Active | 26 | 5.54 | 3.77 | -1.77 | 1.50 |
| Placebo | 28 | 5.21 | 5.25 | 0.04 | 1.00 |

**Supplementary Table 4. Change in baseline in CGI-I per treatment group.**

| Group | N | Baseline Mean Score | Week 8 Mean Score | Mean Change | SD |
| --- | --- | --- | --- | --- | --- |
| Active | 26 | 4 | 2.62 | -1.38 | 0.85 |
| Placebo | 28 | 4 | 4.04 | 0.04 | 1.29 |

**Supplementary Table 5. Change in baseline in Vineland-3 per treatment group.**

| Parameter | Group | N | Baseline Mean Score | Week 8 Mean Score | Mean Change | SD |
| --- | --- | --- | --- | --- | --- | --- |
| Adaptive Behaviour Composite | Active | 26 | 55.35 | 58.00 | 2.65 | 5.07 |
|  | Placebo | 28 | 52.36 | 51.93 | -0.43 | 5.27 |
| Communication | Active | 26 | 57.04 | 58.96 | 1.92 | 5.80 |
|  | Placebo | 28 | 50.82 | 50.50 | -0.32 | 5.19 |
| Daily Living Skills | Active | 26 | 55.62 | 58.62 | 3.00 | 5.48 |
|  | Placebo | 28 | 53.18 | 52.89 | -0.29 | 5.85 |
| Socialisation | Active | 26 | 53.50 | 56.31 | 2.81 | 5.26 |
|  | Placebo | 28 | 53.18 | 52.54 | -0.64 | 7.06 |

**Supplementary Table 6. Change in baseline in SRS-2 per treatment group.**

| Parameter | Group | N | Baseline Mean Score | Week 8 Mean Score | Mean Change | SD |
| --- | --- | --- | --- | --- | --- | --- |
| Total *T*-score | Active | 26 | 76.62 | 74.15 | -2.46 | 5.90 |
|  | Placebo | 28 | 77.54 | 77.68 | 0.14 | 4.23 |
| Social Awareness | Active | 26 | 73.04 | 70.81 | -2.23 | 5.99 |
|  | Placebo | 28 | 75.39 | 74.89 | -0.50 | 5.01 |
| Social Cognition | Active | 26 | 69.77 | 67.04 | -2.73 | 8.80 |
|  | Placebo | 28 | 74.43 | 74.36 | -0.07 | 6.15 |
| Social Communication | Active | 26 | 71.15 | 66.96 | -4.19 | 8.91 |
|  | Placebo | 28 | 70.89 | 69.54 | -1.36 | 5.19 |
| Social Motivation | Active | 26 | 70.73 | 66.58 | -4.15 | 7.06 |
|  | Placebo | 28 | 70.82 | 67.79 | -3.04 | 17.03 |
| Restricted interest and repetitive behaviour | Active | 26 | 70.00 | 67.35 | -2.65 | 8.07 |
|  | Placebo | 28 | 69.29 | 70.61 | 1.32 | 5.68 |

**Supplementary Table 7. Change in baseline in ADAMS per treatment group.**

| Parameter | Group | N | Baseline Mean Score | Week 8 Mean Score | Mean Change | SD |
| --- | --- | --- | --- | --- | --- | --- |
| Total Score | Active | 26 | 50.73 | 30.81 | -19.92 | 10.10 |
|  | Placebo | 28 | 31.79 | 39.29 | 7.50 | 8.77 |
| Manic/hyperactive behaviour | Active | 26 | 6.73 | 7.04 | 0.31 | 4.80 |
|  | Placebo | 28 | 7.04 | 8.46 | 1.43 | 2.57 |
| Depressed mood | Active | 26 | 13.38 | 9.46 | -3.92 | 4.52 |
|  | Placebo | 28 | 5.75 | 8.46 | 2.71 | 4.61 |
| Social avoidance | Active | 26 | 11.92 | 6.65 | -5.27 | 4.94 |
|  | Placebo | 28 | 7.36 | 8.71 | 1.36 | 4.84 |
| General anxiety | Active | 26 | 14.08 | 5.00 | -9.08 | 5.20 |
|  | Placebo | 28 | 8.61 | 9.64 | 1.04 | 4.60 |
| Compulsive behaviour | Active | 26 | 4.62 | 2.65 | -1.96 | 3.18 |
|  | Placebo | 28 | 3.04 | 4.00 | 0.96 | 1.75 |

**Supplementary Table 8. Change in baseline in ASC-ASD-P per treatment group.**

| Parameter | Group | N | Baseline Mean Score | Week 8 Mean Score | Mean Change | SD |
| --- | --- | --- | --- | --- | --- | --- |
| Total Score | Active | 19 | 28.95 | 20.68 | -8.26 | 12.48 |
|  | Placebo | 24 | 20.46 | 25.13 | 4.67 | 15.99 |
| Performance anxiety | Active | 20 | 7.30 | 4.75 | -2.55 | 3.49 |
|  | Placebo | 24 | 4.63 | 5.42 | 0.79 | 4.55 |
| Anxious arousal | Active | 20 | 3.40 | 1.80 | -1.60 | 3.33 |
|  | Placebo | 24 | 2.71 | 2.71 | 0.00 | 3.69 |
| Separation anxiety | Active | 20 | 5.85 | 3.90 | -1.95 | 3.10 |
|  | Placebo | 24 | 4.29 | 5.46 | 1.17 | 3.86 |
| Uncertainty | Active | 20 | 12.30 | 9.20 | -3.10 | 5.24 |
|  | Placebo | 24 | 8.83 | 11.54 | 2.71 | 5.53 |

**Supplementary Table 9. Change in baseline in AFEQ per treatment group.**

| Parameter | Group | N | Baseline Mean Score | Week 8 Mean Score | Mean Change | SD |
| --- | --- | --- | --- | --- | --- | --- |
| Total Score | Active | 26 | 120.65 | 102.81 | -17.85 | 27.07 |
|  | Placebo | 28 | 125.82 | 120.86 | -4.96 | 15.96 |
| Experience of Being a Parent of a Child with autism | Active | 26 | 35.92 | 31.12 | -4.81 | 9.70 |
|  | Placebo | 28 | 35.71 | 34.50 | -1.21 | 5.70 |
| Family Life | Active | 26 | 17.54 | 16.12 | -1.42 | 6.33 |
|  | Placebo | 28 | 22.36 | 21.68 | -0.68 | 6.96 |
| Child Development, Understanding and Social Relationships | Active | 26 | 36.81 | 30.23 | -6.58 | 9.73 |
|  | Placebo | 28 | 37.36 | 35.68 | -1.68 | 7.95 |
| Child Symptoms (feelings and behaviour) | Active | 26 | 30.38 | 25.35 | -5.04 | 10.29 |
|  | Placebo | 28 | 30.39 | 29.00 | -1.39 | 6.93 |

**Supplementary Table 10. Change in baseline in SDSC per treatment group.**

| Parameter | Group | N | Baseline Mean Score | Week 8 Mean Score | Mean Change | SD |
| --- | --- | --- | --- | --- | --- | --- |
| Total Score | Active | 20 | 47.10 | 47.75 | 0.65 | 10.97 |
|  | Placebo | 22 | 53.27 | 53.95 | 0.68 | 11.14 |
| Disorder of initiating and maintaining sleep | Active | 20 | 17.35 | 16.40 | -0.95 | 5.53 |
|  | Placebo | 22 | 19.50 | 19.14 | -0.36 | 6.18 |
| Sleep breathing disorders | Active | 20 | 4.00 | 3.95 | -0.05 | 1.61 |
|  | Placebo | 22 | 4.00 | 4.32 | 0.32 | 1.25 |
| Disorders of arousal | Active | 20 | 3.00 | 3.45 | 0.45 | 1.28 |
|  | Placebo | 22 | 4.00 | 4.45 | 0.45 | 1.44 |
| Sleep-wake transition disorders | Active | 20 | 11.50 | 12.25 | 0.75 | 4.38 |
|  | Placebo | 22 | 13.18 | 14.00 | 0.82 | 4.07 |
| Disorders of excessive somnolence | Active | 20 | 8.70 | 9.40 | 0.70 | 4.09 |
|  | Placebo | 22 | 9.09 | 8.23 | -0.86 | 3.11 |
| Sleep hyperhidrosis | Active | 20 | 2.55 | 2.30 | -0.25 | 1.12 |
|  | Placebo | 22 | 3.50 | 3.82 | 0.32 | 1.86 |

**SUPPLEMENTARY FIGURE LEGENDS**

**Supplementary Fig. 1 Distributions of change from baseline for SRS-2** (a) Distribution of change from baseline in Social Awareness scores at Week 8. (b) Distribution of change from baseline in Social Cognition scores at Week 8. (c) Distribution of change from baseline in Social Communication scores at Week 8. (d) Distribution of change from baseline in Social Motivation scores at Week 8. (e) Distribution of change from baseline in Restricted Interests and Repetitive Behaviour scores at Week 8. (f) Distribution of change from baseline in Total T-scores at Week 8. n = 54, analysed via ANCOVA.

**Supplementary Fig. 2 Distributions of change from baseline for ADAMS** (a) Distribution of change from baseline in Manic/hyperactive scores at Week 8. (b) Distribution of change from baseline in Depressed Mood scores at Week 8. (c) Distribution of change from baseline in Social Avoidance scores at Week 8. (d) Distribution of change from baseline in General Anxiety scores at Week 8. (e) Distribution of change from baseline in Compulsive Behaviour scores at Week 8. (f) Distribution of change from baseline in Total scores at Week 8. n = 54, analysed via ANCOVA.

**Supplementary Fig. 3 Distributions of change from baseline for ASC-ASD-P** (a) Distribution of change from baseline in Performance Anxiety scores at Week 8. (b) Distribution of change from baseline in Uncertainty scores at Week 8. (c) Distribution of change from baseline in Anxious Arousal scores at Week 8. (d) Distribution of change from baseline in Separation Anxiety scores at Week 8. (e) Distribution of change from baseline in Total scores at Week 8. n = 54, analysed via ANCOVA.

**Supplementary Fig. 4 Distributions of change from baseline for AFEQ** (a) Distribution of change from baseline in Experience of Being a Parent with a Child with ASD scores at Week 8. (b) Distribution of change from baseline in Family Life scores at Week 8. (c) Distribution of change from baseline in Child Development, Understanding and Social Relationships scores at Week 8. (d) Distribution of change from baseline in Child Symptoms scores at Week 8. (e) Distribution of change from baseline in Total scores at Week 8. n = 54, analysed via ANCOVA.

**Supplementary Fig. 5 Distributions of change from baseline for SDSC** (a) Distribution of change from baseline in Disorders of Initiating and Maintaining Sleep scores at Week 8. (b) Distribution of change from baseline in Sleep Breathing Disorders scores at Week 8. (c) Distribution of change from baseline in Disorders of Arousal scores at Week 8. (d) Distribution of change from baseline in Sleep-wake Transition Disorders scores at Week 8. (e) Distribution of change from baseline in Disorders of Excessive Somnolence scores at Week 8. (f) Distribution of change from baseline in Sleep Hyperhidrosis scores at Week 8. (g) Distribution of change from baseline in Total scores at Week 8. n = 54, analysed via ANCOVA.
